# Aspartate beta-hydroxylase is a prognostic factor in gallbladder cancer with the function of promoting tumorigenesis and chemoresistance

**DOI:** 10.3389/fendo.2025.1452345

**Published:** 2025-03-05

**Authors:** Luo Yuan, Huang Yunpeng, Li Xiong, Yu Wen, Wang Yongxiang

**Affiliations:** Second Xiangya Hospital, Central South University, Changsha, China

**Keywords:** gallbladder cancer, ASPH, metastasis, immune evasion, drug resistance, prognosis

## Abstract

**Aims:**

Gallbladder cancer is characterized by a dismal prognosis, with a limited number of biological markers currently identified for the carcinogenesis, progression and prognosis of gallbladder cancers (GBCs). The discovery of efficacious biomarkers is crucial for enhancing the prognosis of gallbladder cancer.

**Methods:**

Analysis of RNAseq datasets from gallbladder cancer allowed the identification of differential genes between gallbladder cancer and adjacent tissues. Subsequent application of Mendelian randomization extracted target gene known to promote gallbladder cancer from these differentially expressed genes. Immunohistochemistry was then conducted to evaluate the expression of these target gene in a cohort of 215 patients with gallbladder cancer, utilizing follow-up information to determine their prognostic value. Moreover, single-cell sequencing data of gallbladder cancer elucidated the role of target genes within the immune microenvironment of this cancer type. The Genomics of Therapeutics Response Portal (CTRP) database enabled the assessment of the impact of target genes on the IC50 of chemotherapy drugs. Lastly, network pharmacology and analytical methodologies were employed to investigate the effects of traditional Chinese medicine active ingredients targeting these specific genes.

**Results:**

ASPH expression is notably elevated in gallbladder cancer tissues, correlating with an unfavorable prognosis for patients afflicted with this disease. Results from Mendelian randomization studies suggest that heightened ASPH levels play a significant role in the development of gallbladder polyps and stones, which are established clinical risk factors in gallbladder cancer. Analysis of clinical samples demonstrates a positive association between ASPH expression and indicators of poor differentiation, increased tumor size, advanced TNM stage, lymph node metastasis, and invasion. The single-cell immune microenvironment reveals that ASPH not only enhances the expression of immune checkpoints, namely PDL1 and PVR, in the gallbladder cancer epithelium, resulting in immune evasion, but also triggers epithelial-mesenchymal transition and migration, promoting metastasis. Furthermore, ASPH contributes to heightened tumor drug metabolism, hence raising the IC50 values for gemcitabine and paclitaxel. Utilizing network pharmacology and molecular docking techniques, this study pinpointed six bioactive compounds derived from traditional Chinese medicine with a targeted effect on the ASPH protein, comprising Sebacic acid, Suberic acid, Azelaic acid, Dimelic acid, Succinic acid, and D-Asparaginsaeure.

**Conclusions:**

ASPH plays a role in promoting the development of gallbladder cancer and resistance to chemotherapeutic agents, rendering it a promising target for therapeutic interventions. Active therapeutic compounds targeted on ASPH can be identified among the active ingredients present in traditional Chinese medicine.

## 1 Introduction

Gallbladder cancer is a relatively uncommon malignancy associated with a grim prognosis, burdening regions like Central and South America, Central and Eastern Europe, Japan, and Northern India with a significant disease burden (1, 2). Due to the asymptomatic nature of this type of cancer, early diagnosis presents a challenge, and most patients are already in the advanced stage when detected (3, 4). Most Gallbladder Cancers are of the adenocarcinoma and squamous cell/adenosquamous carcinoma types (5). The poor prognosis of gallbladder cancer underscores the importance of identifying key molecular markers, which not only aid in the diagnosis of gallbladder cancer but also optimize its treatment (6, 7).

ASPH belongs to the α-ketoglutarate-dependent dioxygenase family and can influence Notch and Jagged, thereby playing a role in the cell growth, differentiation, migration, adhesion, and motility of tumors (8–11). Previous studies have indicated a significant association between ASPH and the recurrence of liver cancer, as it facilitates the migration and invasion of liver cancer cells (9, 12–14). In addition, Elevated levels of ASPH expression can also facilitate the advancement of cholangiocarcinoma and pancreatic cancer, ultimately resulting in adverse clinical outcomes (15, 16). Gallbladder cancer is also classified as a type of biliary system tumor; however, research on the association of ASPH with gallbladder cancer is currently lacking.

Thus, we used immunohistochemistry to evaluate the expression of ASPH in surgically resected specimens. Further analysis was conducted to examine the association between ASPH and the clinical characteristics of gallbladder cancer, along with its implications on the prognosis of gallbladder cancer patients. Furthermore, the impact of ASPH on the microenvironment of gallbladder cancer was revealed through single-cell sequencing, and potential drugs targeting ASPH were analyzed using network pharmacology. The main objective of this study is to introduce novel strategies for the diagnosis and treatment of gallbladder cancer.

## 2 Materials and methods

### 2.1 Case selection

This study was pre-approved by the Ethics Committee for Human Research, Central South University. Most GBCs are adenocarcinomas (AC >90%). In contrast, squamous cell/adenosquamous carcinoma (SC/ASC) is rare, representing 1–12% of GBCs. According to the recommendations of the American Joint Committee on Cancer, tumors with a squamous component≥10% were considered to represent adenosquamous carcinomas. A total of 69 SC/ASC samples resulting from surgical resection or biopsy were collected from January 2001 to December 2013. A total of 146 AC samples derived from surgical resection or biopsy at Second Xiangya Hospital and Third Xiangya Hospital were collected between January 2008 and December 2013.

### 2.2 EnVision immunohistochemistry

Four-micrometer-thick sections were cut from routinely paraffin-embedded tissues. The rabbit anti-human ASPH, and HRP-conjugated anti-rabbit second antibody were purchased from Santa Cruz Biotechnology (Santa Cruz, CA, USA). EnVisionTM Detection Kit was purchased from Dako Laboratories (CA, USA). The staining of ASPH was carried out according to the manufacture’s protocol. Briefly, the sections were deparaffinized and then incubated with peroxidase inhibitor (3% H2O2) in the dark for 15 minutes, followed by EDTA-trypsin digestion for 15 minutes. The sections were incubated with primary antibody for 60 minutes, then second antibody for 30 min after being soaked with PBS for 3 × 5 minutes. Solution A was added to the sections for 30 minutes followed by DAB staining and hematoxylin counter-staining. The slides were dehydrated with different concentrations (70%–100%) of alcohol, and soaked in xylene for 3 × 5 minutes and finally mounted with neutral balsam. Ten random fields were examined per section. The percent to positively stained cells relative to the total number of cells was determined. Next, the strength of staining was rated on a scale of 1 to 3. A score of 1 represents little to no positive staining or uncertainly weak staining; a score of 2 represents weak to moderate staining; and a score of 3 represents moderate to strong staining. A section is determined as positive for ASPH when the percent of positively stained cells was ≥ 10% and staining strength ≥2. The few sections where percent positive staining was 5% to 10% and staining strength was 3 were also regarded as positive.

### 2.3 Single-cell data analysis

Data for gallbladder cancer single-cell sequencing was obtained from the GSE201425 dataset. The data was processed and analyzed using the Seurat package to organize the gallbladder cancer sequencing data, and cell annotations were performed using molecular markers from the cellmarker database (http://xteam.xbio.top/CellMarker). Cell grouping was based on the mean expression level of ASPH, with cells above the mean categorized into the high-expression group and those below the mean categorized into the low-expression group. Cell communication differences between the two groups were analyzed using CellChat to investigate ASPH-related cell interactions. Pseudotime analysis of gallbladder cancer epithelium was performed using the Monocle2 package.

### 2.4 Drug sensitivity analysis

A total of 481 small molecules’ IC50 values across 1001 cell lines and their respective mRNA gene expressions were gathered from the Genomics of Therapeutics Response Portal (CTRP). Subsequently, the mRNA expression data was integrated with the drug sensitivity data. Pearson correlation analysis was conducted to assess the relationship between gene mRNA expression levels and drug IC50 values, with P-values adjusted using the false discovery rate (FDR). Based on the expression level of ASPH, cell lines with expression levels higher than the mean were classified into the high-expression group, while those with expression levels lower than the mean were classified into the low-expression group. Subsequently, the differences in drug IC50 between the two groups were analyzed using a rank sum test.

### 2.5 Chinese herbal network pharmacology analysis based on ASPH

Using ETCM (Encyclopaedia of Traditional Chinese Medicine), we extracted the ingredients that target ASPH and identified the herbs that contain these specific ingredients. Subsequently, a network was constructed to interconnect ASPH, these ingredients, and the respective herbs.

### 2.6 Cell culture and cytotoxicity for treatments

The gallbladder cancer cell line (QBC-SD cell) were purchased from the Cell Resource Center of the Shanghai Academy of Biological Sciences and cultured in RPMI-1640 medium supplemented with 10% fetal bovine serum and 1% Penicillin/Streptomycin solution. QBC-SD cellswere cultured in a constant temperature incubator at 37°C and 5% CO2.QBC-SD cells were plated on 96-well plates for 24 h before intervention.

After washing twice with PBS, the QBC-SD were incubated with 0–400 uM Suberic acid(MCE, HY-W015300, United States), 0-400 uM Succinic Acid(MCE, HY-N0420, United States) or 0-1600 nM Azelaic Acid(MCE, HY-B0704, United States) in 100 µL complete medium. We used complete medium containing these drugs to incubate for 1-4 days, these drugs treatment group was incubated with complete medium containing 10% Cell Counting Kit-8 (MCE, HYK0301, United States) at 37°C for 1 h, and the absorption wavelength of 450 nm was detected.

### 2.7 Colony formation experiment and migration assays

QBC-SD cell were seeded on six-well plates with a density of 2000 cells per well. After 24 h, QBC-SD cell were treated with Suberic acid(MCE, HY-W015300, United States), Succinic Acid(MCE, HY-N0420, United States) or Azelaic Acid. After QBC-SD cell were cultured in complete medium for 10 days, they were washed twice with PBS and fixed with 4% paraformaldehyde at 37°C for 15 min. We stained the colonies with crystal violet solution for 15 min, washed it twice with PBS, dried it naturally and took pictures of the colonies with a digital camera (iPhone X).

QBC-SD cell were seeded in uncoated Transwell chambers (Corning, 3422, United States) to detect migration of QBC-SD cell, respectively. 1 × 105 cells were mixed with 200 μL serum-free medium and added to the upper chamber. 600uL 20% FBS medium was added to the lower chamber and incubated for 24 h-48 h. We used cotton swab and slightly wiped the cells in the chamber. QBC-SD cell were fixed with 4% paraformaldehyde at 37°C for 15 min and stained with crystal violet solution for 15 min. After washed twice with PBS, we took pictures and counted them under a microscope.

### 2.8 Western blot

After scraping the cells, the cells were collected by centrifugation at 800 × g. QBC-SD were lysed using 60 μL of RIPA lysis buffer containing a protease inhibitor. After 30 min of lysis on ice, the lysate was centrifuged at 12,000 × g for 10 min to collect the supernatant. Subsequently, 5 × SDS loading buffer was added, and the mixture was denatured at 95°C for 5 min. The electrophoresis conditions used were: 140 V, 45 min. The NC membrane was activated with methanol, followed by transfer at 400 mA for 35 min. The NC membrane was blocked with skimmed milk prepared in TBST at room temperature for 90 min. After incubating the primary antibody overnight and incubating the secondary antibody at room temperature for 50 min. TBST was used for washing three times, ECL solution was used for development, and protein content was analyzed by photography. The primary antibodies were ASPH (Abclonal, A13153, China) with a dilution ratio of 1:1000.

### 2.9 Statistical analysis

Data was analyzed using the statistical package for the Social Sciences Version 13.0 (SPSS 13.0). The inter-relationship of ASPH expression with histology or clinical factors was analyzed using χ2 or Fisher’s exact test. Kaplan-Meier and time series test (log-ranktest) were used for Univariate survival analysis. Cox proportional hazards model was used for multivariate analysis and to determine the 95% confidence interval.KM curves were plotted using the survival package in R, while ROC curves were analyzed and plotted using the pROC package in R.Gene Set Enrichment Analysis (GSEA) was performed using the GSEA software (version 3.0) obtained from the GSEA website. Kyoto Encyclopedia of Genes and Genomes (KEGG) analysis was conducted using the R package clusterProfiler(version 3.14.3) for enrichment analysis. GSEA and KEGG analyses set the minimum gene set size to 5 and the maximum gene set size to 5000, with a p-value of < 0.05 (modifiable as needed) and a false discovery rate (FDR) of < 0.25 (modifiable as needed) considered statistically significant. TwoSampleMR package is used for Mendelian randomization (MR) analysis.

## 3 Results

### 3.1 ASPH promotes the development of gallbladder cancer

**Figure 1:**
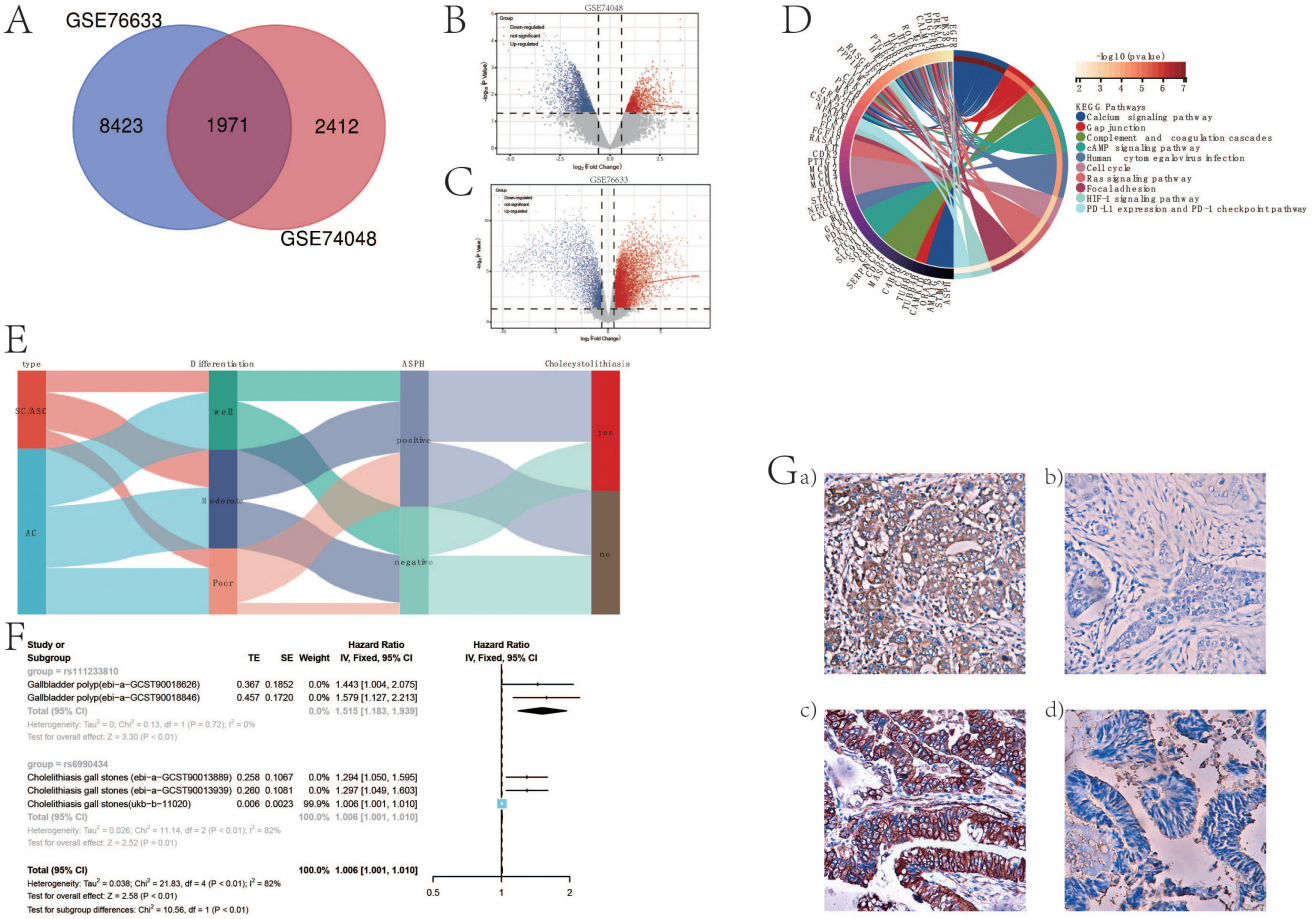
ASPH promotes the occurrence of gallbladder cancer. **(A)** Venn diagram of differentially expressed genes in GSE76633 and GSE74048 datasets. **(B)** Volcano plot of differentially expressed genes in GSE74048. **(C)** Volcano plot of differentially expressed genes in GSE76633. **(D)** Chord diagram of functional enrichment based on protein interaction network of ASPH. **(E)** Sankey diagram of ASPH, pathological type, differentiation, and gallstone information in clinical samples of gallbladder cancer patients. **(F)** Forest plot of Mendelian randomization analysis results on the impact of ASPH on gallbladder polyps and gallstones. **(G)** Distribution features of ASPH in gallbladder cancer: EnVision immunohistochemistry, original magnification ×200. a) Positive expression of ASPH in poorly differentiated squamous cell carcinoma; b) Negative expression of ASPH in well-differentiated squamous cell carcinoma; c) Positive expression of ASPH in moderately differentiated adenocarcinoma; d) Negative expression of ASPH in well-differentiated adenocarcinoma.

Differential gene analysis of gallbladder cancer and adjacent tissues from GSE76633 and GSE74048 revealed an intersection of 1971 differentially expressed genes (**Figure 1A**) (specific details in venn_result.txt). ASPH demonstrated high expression within these 1971 genes, as illustrated in specific figures (**Figures 1B, C**). Subsequently, a protein interaction network associated with ASPH was constructed using these 1971 differential genes (details in **Supplementary Figure S1**) and subsequently enriched through ASPH’s protein interaction network. The analysis revealed that ASPH influences crucial processes such as the cell cycle, tumor immunity, and cell adhesion, which are integral in gallbladder cancer development (**Figure 1D**).

In order to demonstrate the clinical impact of ASPH on gallbladder cancer, this study included 215 patients with bile duct cancer (69 cases of SC/ASC and 146 cases of AC). Immunohistochemistry was used to assess the expression of ASPH in these patients, with no statistical difference in the positivity rate between SC/ASC and AC groups (**Table 1**). The percentage of cases exhibiting lymph node metastasis and invasion was significantly higher in the SCs/ASCs compared to the AC (P<0.05). Correlation analysis between ASPH and clinical characteristics of gallbladder cancer revealed that ASPH impacts the differentiation, tumor size, TNM stage, and invasion of gallbladder cancer (details in **Supplementary Table S1**).

**Table 1:** Comparison of gallbladder SC/ASC and AC clinicopathological features and ASPH expression status.

Clinicopathological characteristics	SC/ASC (n=69)	AC (n=146)	X2	P value
Gender, n (%)				
Male	25 (36.2)	61 (41.8)	0.601	0.438
Female	44 (63.8)	85 (58.2)		
Age, n (%)				
≤45 years	3 (4.3)	20 (13.7)	4.289	0.038
>45 years	66 (95.7)	126 (86.3)		
Differentiation, n (%)				
Well	19 (27.5)	51 (34.9)	2.235	0.308
Moderate	33 (47.8)	54 (37.0)		
Poor	17 (24.6)	41 (28.1)		
Maximum tumor diameter, n (%)				
≤3 cm	39 (56.5)	90 (61.6)	0.512	0.474
>3 cm	30 (43.5)	56 (38.4)		
Cholecystolithiasis, n (%)				
(-)	31 (44.9)	78 (53.4)	1.353	0.245
(+)	38 (55.1)	68 (46.6)		
TNM stages, n (%)				
I+II	29 (42.0)	77 (52.7)	2.151	0.143
III + IV	40 (58.0)	69 (47.3)		
Lymph node metastasis, n (%)				
(-)	27 (39.1)	80 (54.8)	4.599	0.032
(+)	42 (60.9)	66 (45.2)		
Locoregional invasion, n (%)				
(-)	24 (34.8)	72 (49.3)	4.004	0.045
(+)	45 (65.2)	74 (50.7)		
Surgical methods, n (%)				
Radical	27 (39.1)	75 (51.4)	3.002	0.223
Palliative	28 (40.6)	50 (34.2)		
Without resection	14 (20.3)	21 (14.4)		
ASPH				
(-)	25 (36.2)	70 (47.9)	2.607	0.106
(+)	44 (63.8)	76 (52.1)		

Subsequently, utilizing a Sankey diagram analysis, it was found that ASPH-positive patients had a higher proportion of poor differentiation (strong proliferative ability) and a higher proportion of patients with stones. Gallbladder polyps and gallstones are risk factors for gallbladder cancer. Using Mendelian randomization and meta-analysis, it was discovered that ASPH promotes the occurrence of gallbladder polyps and gallstones. The formation of gallbladder polyps is related to abnormal differentiation of gallbladder epithelium, indicating a decrease in the degree of differentiation of the gallbladder epithelium. This further confirms that ASPH affects the differentiation process of gallbladder cancer. Conversely, immunohistochemical results showed that the majority of ASPH-positive reactions were localized in the cytoplasm of the SC/ASC and AC. Furthermore, ASPH expression was positive in poorly differentiated squamous cell, and negative in well-differentiated squamous cell. The expression of ASPH was positive in moderately differentiated adenocarcinoma and negative in well-differentiated adenocarcinoma. In conclusion, the above information indicates that ASPH can promote the development of gallbladder cancer.

### 3.2 ASPH can act as prognosis predictor of gallbladder cancer

**Figure 2:**
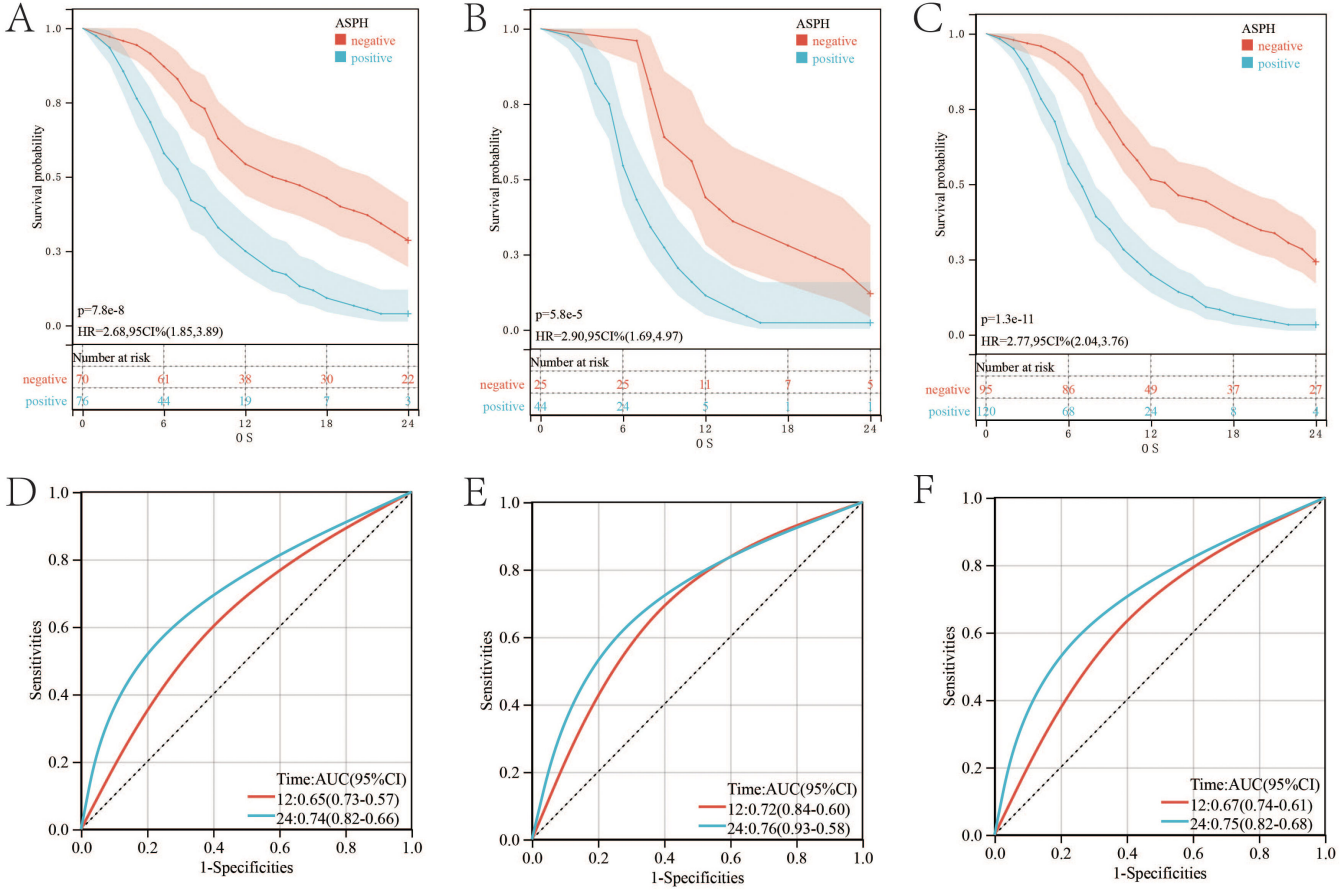
ASPH expression and survival in patients with gallbladder cancer. **(A–C)** Kaplan–Meier plots of overall survival in SC/ASC, AC and all gallbladder patients. **(D–F)** ROC of Diagonal segments is produced by ASPH in SC/ASC, AC and all gallbladder patients.

In SC/ASC patients, ASPH positive expression has poor prognosis compared with ASPH negative expression (p value=7.8e-8; HR=2.68) (**Figure 2A**). ROC is adopted to evaluate the effect of ASPH as a prognosis predictor. The AUC of ROC is 0.65 (95%CI: 0.57–0.73) for ASPH as a 1 year prognosis predictor and 0.74 (95%CI: 0.66–0.82) as a 2 year prognosis predictor, respectively (**Figure 2D**).

The prognosis of AC patients with ASPH positive expression is worse than that of patients with ASPH negative expression. (p value=5.8e-5; HR=2.9) (**Figure 2B**). The AUC values for one and two years are 0.72 (95%CI: 0.6–0.84) and 0.76 (95%CI: 0.58–0.93) respectively (**Figure 2E**).

Prognostic trends were similar among all patients with gallbladder cancer (p value=1.3e-11;HR=2.77) (**Figure 2C**). The AUC values for one and two years are 0.67 (95%CI: 0.61–0.74) and 0.75 (95%CI: 0.68–0.82) respectively. (**Figure 2F**).

### 3.3 The impact of ASPH on the microenvironment of gallbladder cancer

**Figure 3:**
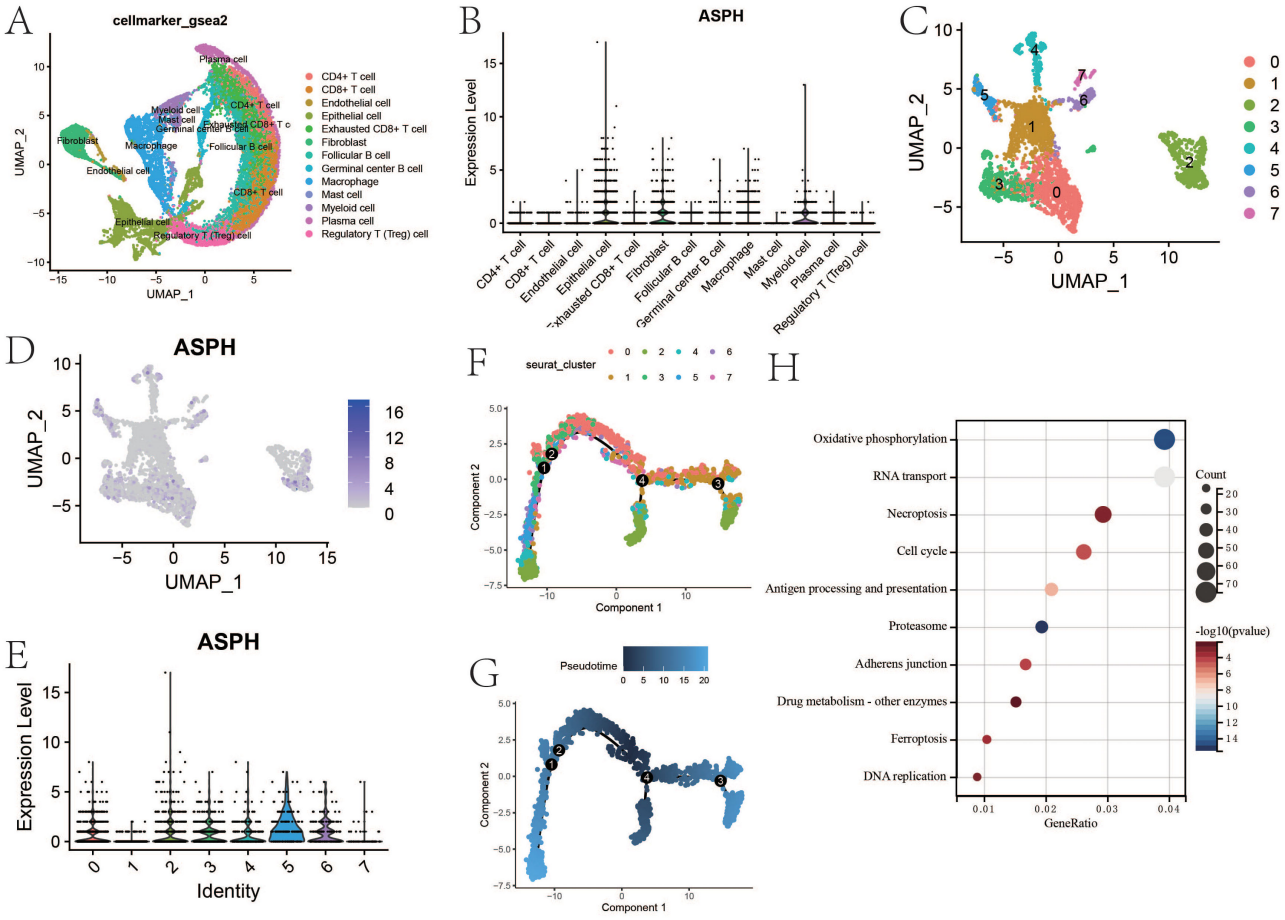
The feature of ASPH expression in gallbladder cancer. **(A)** The uamp of cells in gallbladder cancer. **(B)** The expression of ASPH in different types of cell. **(C)** The uamp of subgroups of epithelial. **(D)** The UAMP of expression of ASPH in subgroups of epithelial. **(E)** The expression of ASPH in subgroups of epithelial. **(F, G)** Cell trajectory of gallbladder cancer epithelial. **(H)** KEGG based on DEGs (cluster 2 vs other clusters).

According to the single-cell dataset, there are lots of immunosuppressive cells including exhausted CD8^+^ T cell and regulatory T cell in gallbladder cancer (**Figure 3A**). The expression of ASPH is higher in epithelial, fibroblast, myeloid cell and macrophage (**Figure 3B**). Epithelial consists of six clusters, and 2 cluster has the highest expression of ASPH (**Figures 3C-E**). According to pseudotime analysis, 2 cluster of epithelial is in the position of newest subgroup and initial subgroup, which means that 2 cluster of epithelial is similar to that of tumor stem cells (**Figures 3F, G**), which promote the development of gallbladder tumor. According to KEGG based on DEGs (cluster 2 vs other clusters), cell cycle, antigen processing and presentation, adherens junction and drug metabolism are significant in cluster 2 epithelial (**Figure 3H**). The cell cycle pathway is corresponding to the results of pseudotime analysis.

**Figure 4:**
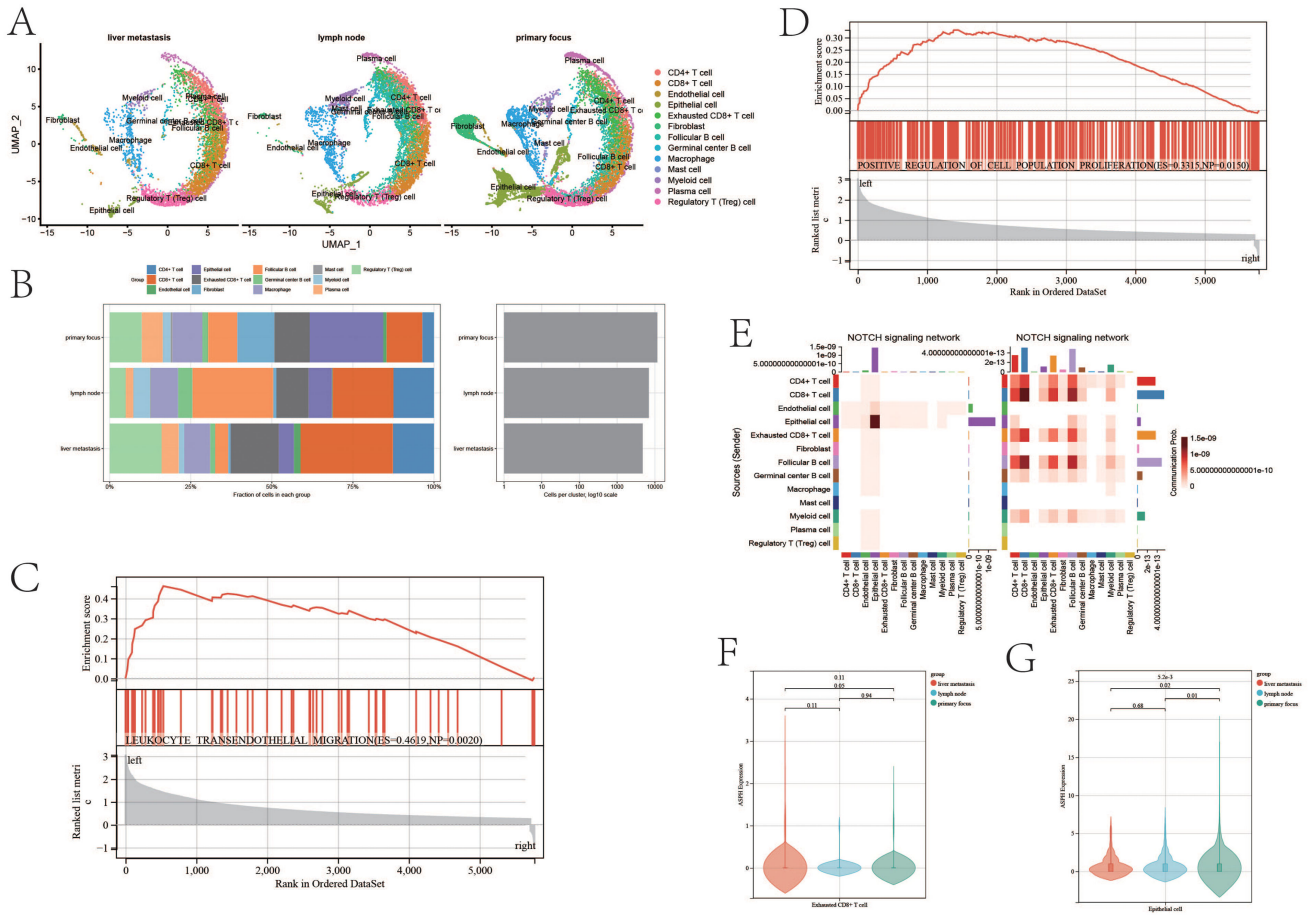
ASPH promotes progression of gallbladder cancer. **(A)** The uamp of cells in different sites of gallbladder cancer. **(B)** Cell proportion of metastatic site, primary site and lymph nodes. **(C, D)** GSEA based on ASPH expression. **(E)** The heatmap of notch pathway (left:high expression of ASPH group, right: low expression of ASPH group). **(F, G)** The ASPH expression of metastatic site, primary site and lymph nodes in Exhausted T cells and epithelial cells.

There is significant difference in tumor environment among **Figure 4A**. The proportions of various immune cells in the immune microenvironment of gallbladder cancer are shown in **Figure 4B**. The proportion of Exhausted T cells is 0.11 in the primary site, 0.1 in the lymph nodes, and 0.15 in the metastatic site. The proportions of Treg cells in the primary site, lymph nodes and metastatic sites were 0.099, 0.05 and 0.16 respectively. Exhausted T cells and Treg cells can lead to immunological tolerance in tumor development.

According to the result of GSEA, compared with low expression of ASPH, high expression of ASPH promotes leukocyte transendothelial migration (ES=0.4619, p value=0.002) and cell proliferation (ES=0.3315,p value=0.015) (**Figures 4C, D**). The results are consistent with the above cell proportion results. The notch pathway is mainly activated in epithelial instead of immune cell in high expression group of ASPH (**Figure 4E**). In Exhausted T cells, the expression of ASPH is highest in liver metastasis of gallbladder cancer, and the expression of ASPH is higher in primary focus of gallbladder cancer than that in lymph node of gallbladder cancer (**Figure 4F**). In epithelial, the expression of ASPH was highest in primary focus of gallbladder cancer, and the expression of ASPH is higher in lymph node of gallbladder cancer than that in liver metastasis of gallbladder cancer (**Figure 4G**). To sum up, ASPH can lead to the proliferation of tumor epithelium and depletion of immune cells in gallbladder cancer patients.

### 3.4 ASPH inhibits cancer immune response in patients with gallbladder cancer

According to the result of GSEA, compared with low expression of ASPH, high expression of ASPH inhibits T cell receptor signaling pathway (ES=-0.2306, p value<0.001) and B cell receptor signaling pathway (ES=-0.2894, p value<0.001) (**Supplementary Figures S2A, B**). It means ASPH can inhibit the function of immune cells to clear tumor cells. According to the result of Cellchat, compared with low expression of ASPH, high expression of ASPH significantly downregulates the MHC1 signaling pathway in the tumor microenvironment of gallbladder cancer (**Supplementary Figure S2C**). Correspondingly, high expression of ASPH significantly reduces the IL1 signaling pathway, complement signaling pathway and IFNII signaling pathway in the tumor microenvironment of gallbladder cancer (**Supplementary Figures S2D-F**). Therefore, high ASPH expression in gallbladder cancer may lead to immune tolerance.

**Figure 5:**
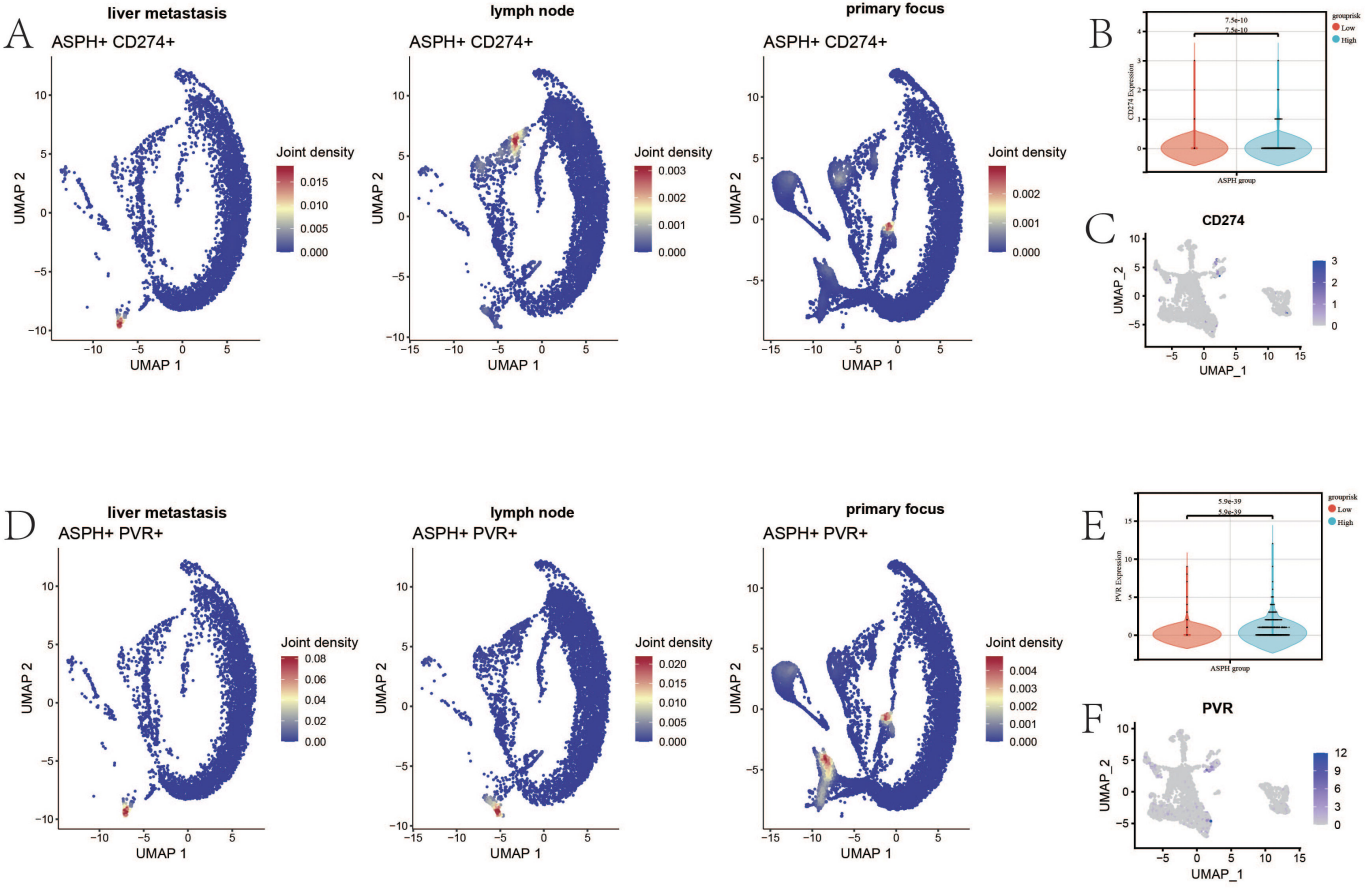
The relationship between ASPH and immune checkpoints. **(A)** The UAMP of co-expression of ASPH and CD274 (PDL1) in metastatic site, primary site and lymph nodes. **(B)** The boxplot of expression of CD274 in high expression of ASPH group and low expression of ASPH group. **(C)** The UAMP of expression of CD274 in subgroups of epithelial. **(D)** The UAMP of co-expression of ASPH and PVR (CD155) in metastatic site, primary site and lymph nodes. **(E)** The boxplot of expression of PVR in high expression of ASPH group and low expression of ASPH group. **(F)** The UAMP of expression of PVR in subgroups of epithelial.

PDL1 (CD274) - PD1 and TIGIT - CD155 (PVR) are common immune checkpoint pathways in tumors, through which tumor cells can inhibit the function of T cells by expressing CD274 and PVR. CD274 and ASPH are co-expressed in endothelial cells in primary lesions, liver metastases, and lymph nodes (**Figure 5A**). In the epithelium of gallbladder cancer, the expression of epithelial CD274 is higher in the ASPH high-expression group (p value = 7.5e-10) (**Figure 5B**). The CD274 expression is mainly found in cluster 2 and cluster 6 (**Figure 5C**). PVR and ASPH are co-expressed in endothelial cells in primary lesions, lymph nodes, and liver metastases (**Figure 5D**). In the epithelium of gallbladder cancer, the expression of epithelial PVR is higher in the ASPH high expression group (p value = 5.9e-39) (**Figure 5E**), and CD274 expression is mainly found in cluster 5 and cluster 6 (**Figure 5F**). Therefore, inhibiting ASPH expression in gallbladder cancer is beneficial for enhancing the response rate to immune therapy for gallbladder cancer.

### 3.5 ASPH promotes metastasis of gallbladder cancer

**Figure 6:**
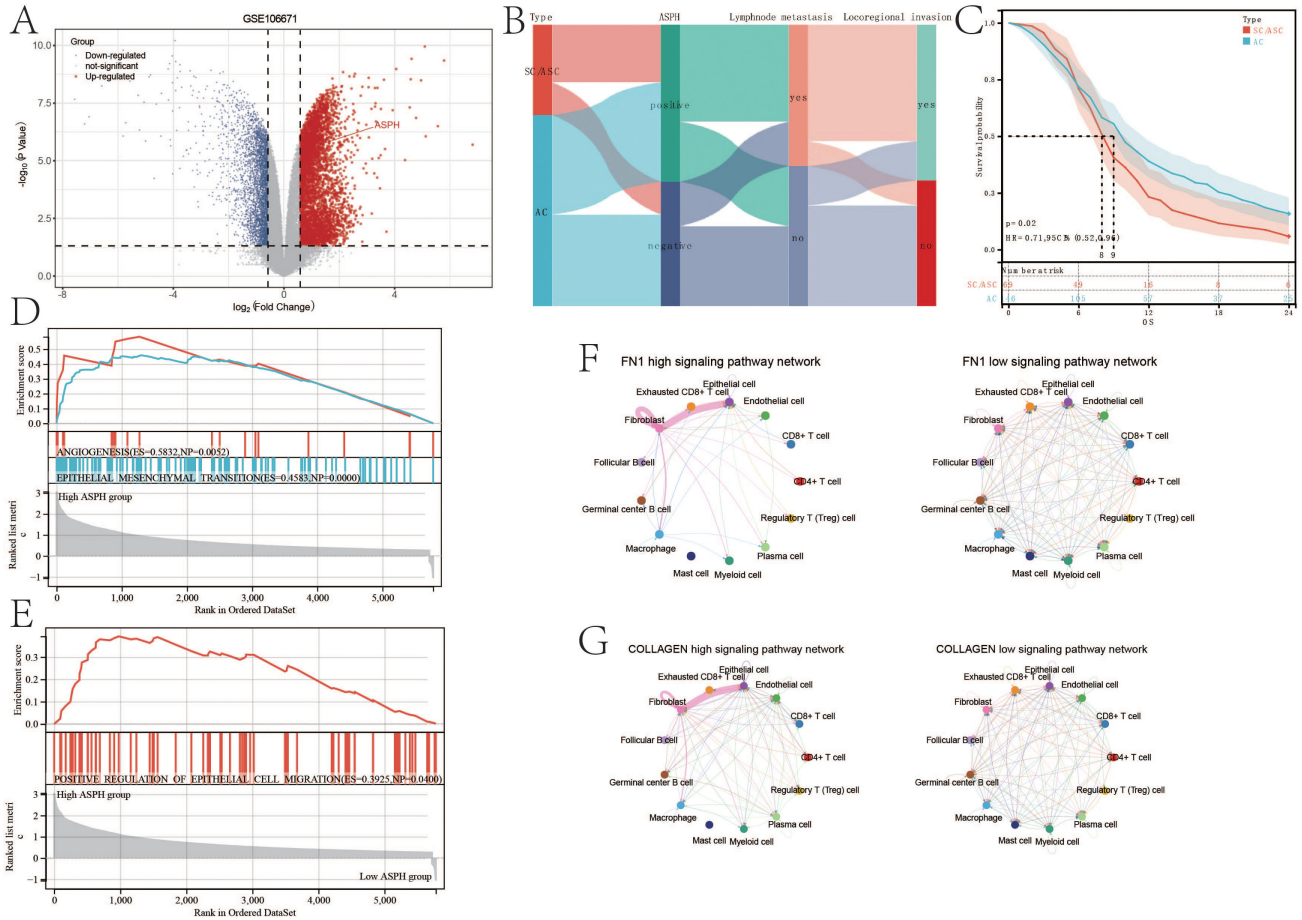
ASPH promotes the metastasis of gallbladder cancer. **(A)** Volcano plot of differentially expressed genes in GSE106671. **(B)** Sankey diagram of ASPH, pathological type, lymph node metastasis and locoregional invasion information in clinical samples of gallbladder cancer. **(C)** Prognostic comparison of different pathological types of gallbladder cancer (SC/ASC and AC). **(D, E)** Gene Set Enrichment Analysis (GSEA) on metastasis based on ASPH expression. **(F, G)** Circular representation of the FN1, VISFATIN, COLLAGEN, and SPP1 signaling pathways (left: high expression of ASPH group, right: low expression of ASPH group).

Differential analysis of gallbladder cancer parental cells and metastatic cells from GSE106671 revealed increased ASPH expression in metastatic gallbladder cancer cells (**Figure 6A**). Findings from 275 gallbladder cancer patients in this study demonstrated that ASPH-positive individuals had a higher incidence of lymph node metastasis and locoregional invasion when compared to ASPH-negative individuals. Furthermore, ASPH-positive patients were more prevalent in SC/ASC cases than in AC cases (**Figure 6B**). Correspondingly, the survival curve outcomes suggested a more favorable prognosis for SC/ASC patients than for AC patients (**Figure 6C**).

According to the result of GSEA, compared with low expression of ASPH, high expression of ASPH inhibits ANGIOGENESIS (ES=0.5832,NP=0.0052), EPITHELIAL_MESENCHYMAL_TRANSITION (ES=0.4583,NP=0.0000) and POSITIVE_REGULATION_OF_EPITHELIAL_CELL_MIGRATION (ES=0.3925, NP=0.0400) (**Figures 6D, E**). According to the result of Cellchat, compared with low expression of ASPH, high expression of ASPH significantly enhances the FN1 signaling pathway and COLLAGEN signaling pathway between epithelial and fibroblast in the tumor microenvironment of gallbladder cancer (**Figures 6F, G**). High expression of ASPH significantly enhances the VISFATIN signaling pathway among epithelial, fibroblast, myeloid cell and macrophage and SPP1 signaling pathway among epithelial, fibroblast and macrophage in the tumor microenvironment of gallbladder (**Supplementary Figures S3A, B**). The four pathways play important roles in promoting metastasis of gallbladder cancer.

### 3.6 Nomogram of gallbladder cancer

The results of univariate Cox regression analysis showed that, in SC/ASC patients, factors such as differentiation, tumor size, TNM stage, lymph node metastasis, invasion, surgical procedure, gallstones, and positive ASPH expression were significantly associated with average survival time (P<0.01; **Table 2**); while in AC patients, differentiation, tumor size, TNM stage, lymph node metastasis, invasion, surgical procedure, and ASPH were significantly associated with average survival time (P<0.01; **Table 2**). Multivariate Cox analysis revealed that differentiation, tumor size, gallstones, TNM stage, invasion, lymph node metastasis, surgical procedure, and positive ASPH expression were correlated with overall survival in SC/ASC (**Table 3**); differentiation, tumor size, TNM stage, lymph node metastasis, invasion, surgical procedure, and positive ASPH expression were correlated with overall survival in AC patients. These findings indicate that the positive expression of ASPH is a risk factor for AC patients (**Table 4**). Taken together, these results suggest that, in both SC/ASC and AC patients, factors such as differentiation, tumor size, TNM stage, invasion, lymph node metastasis, surgical procedure, and positive ASPH expression are independent prognostic factors for gallbladder cancer, while gallstones are solely an independent risk factor for SC/ASC.

**Table 2:** Relationship between ASPH expression, clinicopathological characteristics and average survival of SC/ASC and AC patients.

Clinicopathological characteristics	SC/ASC				AC			
	Sample (n)	Average survival (month)	χ2	P value	Sample (n)	Average survival (month)	χ2	P value
Differentiation								
Well	19	13.68 (5–24)	20.815	0	51	16.69 (5–24)	55.1	0
Moderately	33	11.58 (4–24)			54	12.33 (2–24)		
Poorly	17	6.12 (2–14)			41	6.49 (1–24)		
Tumor size								
≤3cm	30	14.57 (6–24)	21.493	0	90	14.6 (1–24)	23.2	0
>3cm	39	7.44 (2–24)			56	8.38 (1–24)		
Gallstones								
No	31	8.26 (3–18)	7.125	0.008	78	12.19 (2–24)	0	0.98
Yes	38	12.90 (2–24)			68	12.24 (1–24)		
TNM stage								
I+II	29	16.31 (3-24)	46.137	0	77	16.99 (3–24)	87.5	0
III+IV	40	6.83 (2–14)			69	6.88 (1–24)		
Lymph node metastasis								
No	27	16.04 (3–24)	29.663	0	80	16.35 (2–24)	71.4	0
Yes	42	7.45 (2-15)			66	7.2 (1–24)		
Invasion								
No	24	17.25 (3-24)	36.974	0	72	18.08 (4–24)	125	0
Yes	45	7.38 (2-20)			74	6.5 (1–14)		
Surgery								
Radical	27	16.93 (5-24)	54.66	0	75	17.84 (6-24)	150	0
Palliative	28	7.32 (2-12)			50	6.86 (1–14)		
Biopsy	14	6.00 (4-8)			21	4.86 (1–9)		
ASPH								
(-)	25	15.46 (7-24)	16.721	0	70	15.44 (2-24)	28.8	0
(+)	44	7.96 (2-24)			76	9.24 (1-24)		

**Table 3:** Multivariate Cox regression analysis of survival rate in SC/ASC patients.

Groups	Factors	B	SE	Wald	P	RR	95% CI	
							Lower	Upper
Differentiated degree	Well/moderately/poorly	0.543	0.211	6.591	0.01	1.721	1.137	2.604
Tumor size	≤3 cm/>3 cm	0.723	0.32	5.091	0.024	2.06	1.1	3.858
Gallstone	No/yes	0.548	0.264	4.3	0.038	1.731	1.031	2.9062
TNM stage	I+II/III+IV	0.855	0.383	4.98	0.026	2.35	1.11	4.979
Lymph node metastasis	No/yes	1.038	0.427	5.919	0.015	2.823	1.223	6.512
Invasion	No/yes	1.554	0.554	7.871	0.005	4.729	1.597	14.004
Surgery	Radical/Palliative/Biopsy	0.796	0.288	7.649	0.006	2.217	1.261	3.8984
ASPH	(-)/(+)	0.994	0.322	9.509	0.002	2.701	1.436	5.08

**Table 4:** Multivariate Cox regression analysis of survival rate in AC patients.

Groups	Factors	B	SE	Wald	P	RR	95% CI	
							Lower	Upper
Differentiated degree	Well/moderately/poorly	0.46	0.17	7.757	0.005	1.584	1.146	2.189
Tumor size	≤3 cm/>3 cm	0.81	0.4	3.972	0.046	2.239	1.013	4.945
Gallstone	No/yes	0.32	0.21	2.278	0.131	1.377	0.909	2.087
TNM stage	I+II/III+IV	0.93	0.41	5.133	0.023	2.528	1.133	5.638
Lymph node metastasis	No/yes	0.82	0.34	5.809	0.016	0.279	1.166	4.453
Invasion	No/yes	1.64	0.44	13.88	0	5.165	2.177	12.26
Surgery	Radical/Palliative/Biopsy	0.69	0.27	6.791	0.009	1.997	1.187	3.36
ASPH	(-)/(+)	0.69	0.28	6.058	0.014	1.988	1.15	3.436

**Figure 7:**
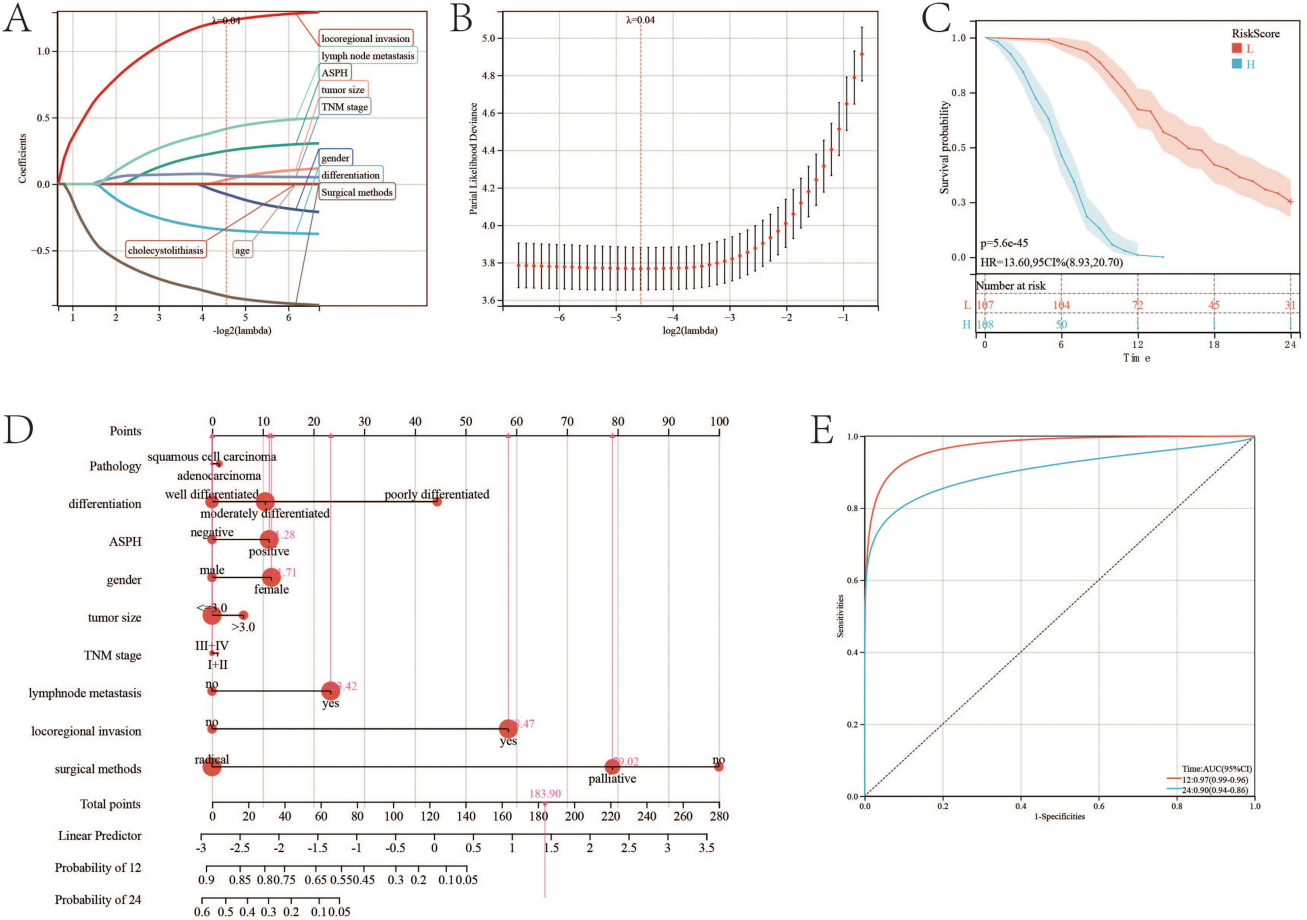
Nomogram of gallbladder cancer based on ASPH. **(A)** Changes in LASSO regression variable coefficients with the variation of the alpha parameter. **(B)** Changes in LASSO regression mean absolute error. **(C)** Kaplan–Meier plots of overall survival in gallbladder cancer patients with high-risk score and low risk score. **(D)** Nomogram of gallbladder cancer patients. **(E)** ROC of Diagonal segments are produced by nomogram riskscore in all gallbladder cancer patients.

To construct a comprehensive prognosis model, lasso regression is adopted to select variables to construct the prognostic nomogram of gallbladder cancer. Similar to cox regression, pathology, differentiation, tumor size, TNM stage, lymph node metastasis, locoregional invasion, surgical methods and ASPH are significant to prognosis of gallbladder cancer (**Figures 7A, B**). The nomogram for gallbladder cancer was constructed [C-index:0.859, 95%CI(0.842-0.877), pvalue<0.001] (**Figure 7D**). High riskscore has poor prognosis (p value=5.6e-45;HR=13.6) (**Figure 7C**). The AUC of ROC of the nomogram is 0.97 (95%CI: 0.96–0.90) for ASPH as a 1 year prognosis predictor and 0.9 (95%CI: 0.86–0.94) as a 2 years prognosis predictor (**Figure 7E**).

### 3.7 ASPH can act as a therapeutic target for drug treatment of gallbladder

**Figure 8:**
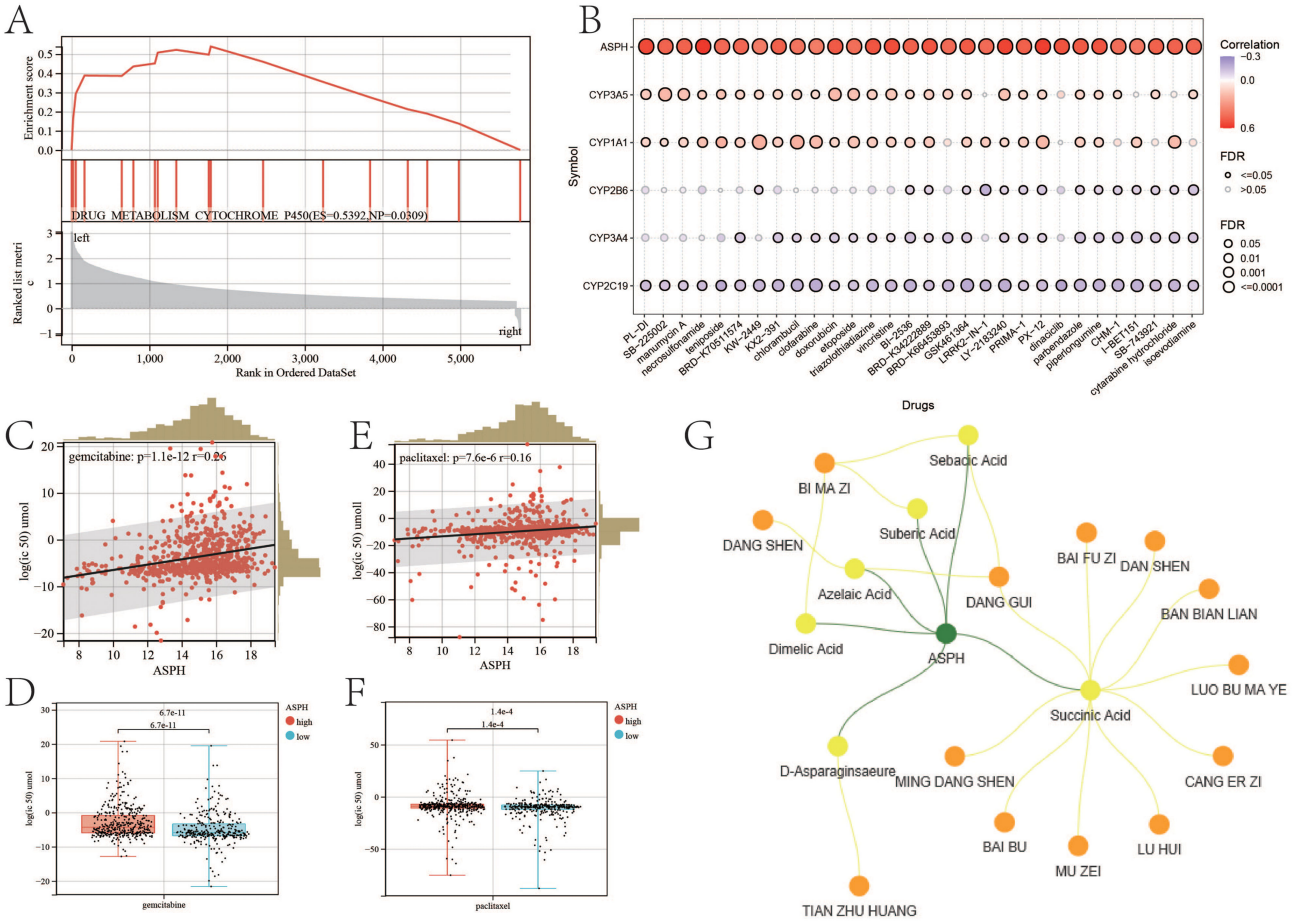
ASPH leads to drug resistance. **(A)** GSEA about drug metabolism based on ASPH expression. **(B)** The correlation between IC50 of drugs and expression of ASPH. **(C)** The correlation plot between Log2 (IC50) of gemcitabine and expression of ASPH. **(D)**The boxplot of Log2 (IC50) of gemcitabine in high expression of ASPH group and low expression of ASPH group. **(E)** The correlation plot between Log2 (IC50) of paclitaxel and expression of ASPH. **(F)** The boxplot of Log2(IC50) of paclitaxel in high expression of ASPH group and low expression of ASPH group. **(G)** Network pharmacology targeting ASPH in Chinese herbal medicine.

According to the result of GSEA, compared with low expression of ASPH, high expression of ASPH promotes DRUG_METABOLISM_CYTOCHROME_P450 (ES=0.5392, NP=0.0309) (**Figure 8A**). Cytochrome P450 (CYP450) are a group of enzymes encoded by the P450 genes (CYP1A1, CYP2B6, CYP2C19, CYP3A4, CYP3A5) and responsible for the metabolism of most drugs in clinical practice, subsequently reducing the therapeutic concentration, which may cause treatment failure. According to the relationship between IC50 and gene expression, ASPH makes difference to IC50 of amounts of drug (**Figure 8B**) (details in Supplementary DrugIC50.xlsx). The results indicate that high expression of ASPH results in drug tolerance in tumor patients. Gemcitabine and paclitaxel are commonly used chemotherapy drugs for gallbladder cancer. In tumor cells, expression of ASPH is positive with log2 (IC50) of gemcitabine (p=1.1e-12, r=0.26) (**Figure 8C**), and the log2 (IC50) of gemcitabine is higher in high expression ASPH tumor cells than that in low expression ASPH tumor cells (p=6.7e-11) (**Figure 8D**). Expression of ASPH is positive with log2 (IC50) of paclitaxel (p=7.6e-6, r=0.16) (**Figure 8E**), and the log2 (IC50) of paclitaxel is higher in high expression ASPH tumor cells (p=1.4e-4) (**Figure 8F**).

Based on the target of ASPH, a network containing herbs and ingredients was constructed (**Figure 8G**). There are six ingredients (sebecic acid, suberic acid, azelaic acid, dimelic acid, succinic acid and D-asparaginsaeure) targeting on ASPH. Dang shen (Codonopsis pilosula), Dang gui(Angelica sinensis) and Bi ma zi (Ricinus communis L.) contain most of these ingredients. Tian zu huan (Bambusa textilis) contains D-asparaginsaeure.

The drug-likeness of each ingredient was estimated by calculating pharmacokinetic parameters using models from the Pipeline Pilot ADMET collection. These parameters included aqueous solubility, blood-brain barrier penetration, CYP450 2D6 inhibition, hepatotoxicity, human intestinal absorption and plasma protein binding. The drug-likeness values for Sebacic acid, Suberic acid, Azelaic acid, Dimelic acid, Succinic acid and D-Asparaginsaeure are 0.602, 0.612, 0.610, 0.608, 0.530 and 0.367, respectively (details in **Supplementary Table S2**).

### 3.8 Molecular docking between ASPH and ingredients

**Figure 9:**
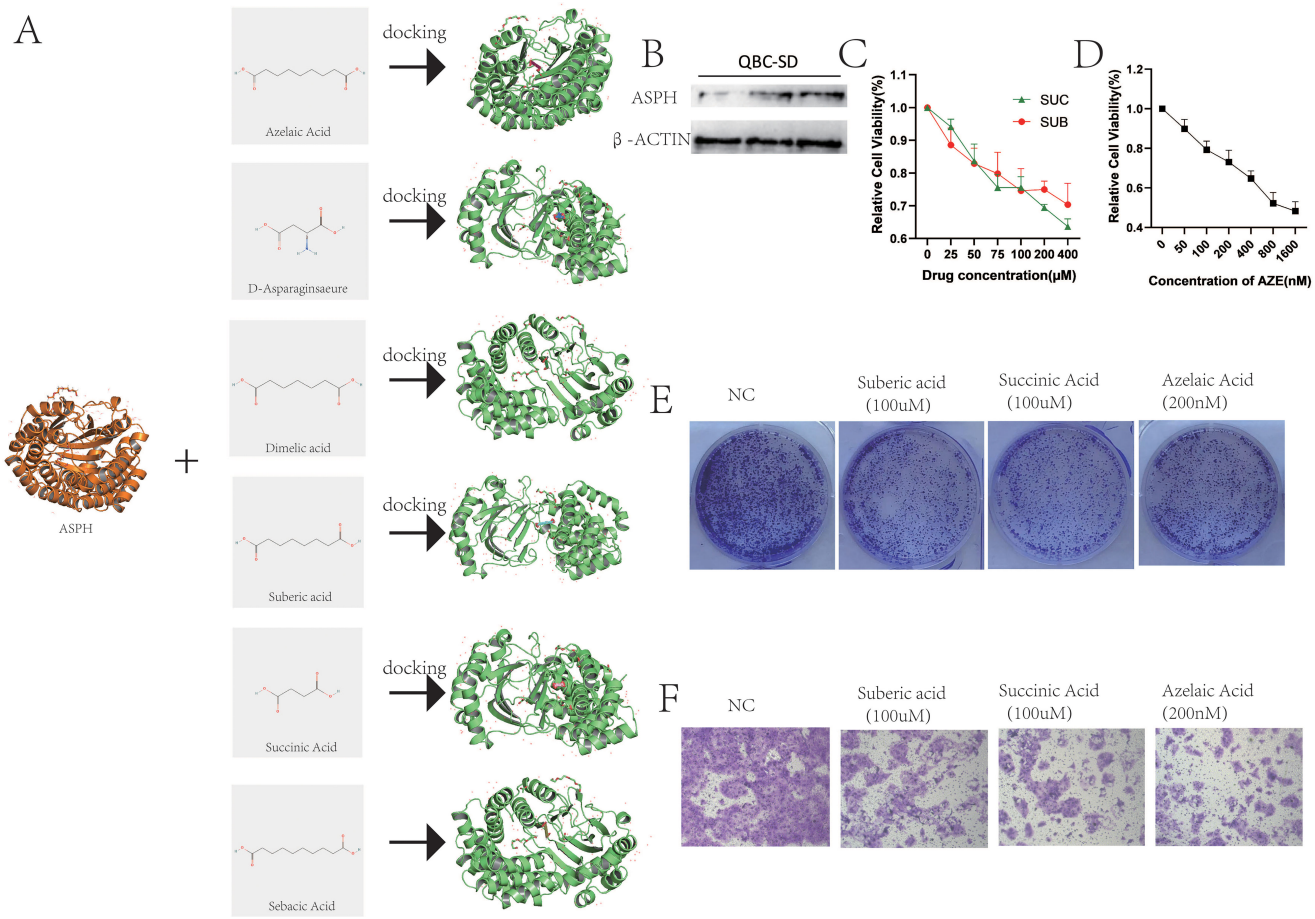
Functional validation of ingredients targeting ASPH. **(A)** Molecular docking between ASPH protein and Sebacic acid, Suberic acid, Azelaic acid, Dimelic acid, Succinic acid, and D-Asparaginsaeure. **(B)** The protein of ASPH in QBC-SD. **(C)** QBC-SD cell were treated with 0–400 uM Suberic acid or 0-400 uM Succinic Acid for 1–4 days, and cytotoxicity was measured by CCK-8 assay. **(D)** QBC-SD cell were treated with 0-1600 nM Azelaic Acid for 1–4 days, and cytotoxicity was measured by CCK-8 assay. **(E)** QBC-SD cell were seeded in 6-well plates and treated with PBS,100 uM Suberic acid, 100 uM Succinic Acid or 200nM Azelaic Acid for 10 days. **(F)** QBC-SD cell were treated with PBS,100 uM Suberic acid, 100 uM Succinic Acid or 200nM Azelaic Acid and cell migration was analyzed by transwell chamber assays.

This study utilized molecular docking to analyze the interactions between the ASPH protein and Sebacic acid, Suberic acid, Azelaic acid, Dimelic acid, Succinic acid, and D-Asparaginsaeure. The results of the molecular docking analysis are presented in **Figure 9A**. Affinity serves as a crucial determinant in drug design, influencing the binding efficacy between drug molecules and target proteins. Specifically, the affinity values between ASPH and Suberic acid, Sebacic acid, Azelaic acid, Succinic acid, D-Asparaginsaeure and Dimelic acid are -6.056 (kcal/mol), -6.647 (kcal/mol), -6.404 (kcal/mol), -4.125 (kcal/mol), -4.416 (kcal/mol), and -5.595 (kcal/mol) respectively (**Table 5**).

**Table 5:** Results of molecular docking.

Docking	POSE	Affinity (kcal/mol)	dist from best mode RMSD l.b.	dist from best mode RMSD u.b.
Suberic acid_ASPH	1	-6.056	0	0
Sebacic Acid_ASPH	1	-6.647	0	0
Azelaic Acid_ASPH	1	-6.404	0	0
Succinic Acid_ASPH	1	-4.125	0	0
D-Asparaginsaeure_ASPH	1	-4.416	0	0
Dimelic acid_ASPH	1	-5.595	0	0

### 3.9 Functional validation of ingredients

The gallbladder cancer cell line QBC-SD expresses the ASPH protein (**Figure 9B**). Therefore, we used QBC-SD cells to examine the effects of suberic acid, azelaic acid, and succinic acid. The experimental results showed a significant decrease in cell viability of QBC-SD cells with increasing concentrations of suberic acid, azelaic acid, and succinic acid (**Figures 9C, D**). Correspondingly, colony formation assays confirmed that these three drugs significantly inhibited the proliferation of QBC-SD cells (**Figure 9E**). Additionally, transwell assays showed that these three drugs markedly suppressed the migration of QBC-SD cells (**Figure 9F**). These results suggest that suberic acid, azelaic acid, and succinic acid are potential therapeutic agents for gallbladder cancer, and their effects on gallbladder cancer cells are highly consistent with the function of ASPH. However, further research is needed to investigate their mechanisms of action on ASPH.

## 4 Discussion

The clinical and pathological characteristics of SC/ASC primarily stem from individual case reports and limited case series analyses. Further research is essential to comprehensively understand the distinctions between rare SC/ASC tumors and typical adenocarcinomas. The reported incidence of squamous differentiation is 1-12% in gallbladder malignancies (4, 6) and in the present study 4.34% SC/ASC were observed. A previous study identified that the occurrence of SC/ASC is predominant in females (F/M, 3.8) (17), however in the present study there was no significant difference (F/M, 1.4). In previous studies, it has been demonstrated that the proliferation of SC occurs at a higher rate than AC, whereas the prevalence of squamous tumors is less frequent with lymph node metastasis (18, 19). Observations from the present study revealed the percentage of cases with a patient age of >45 years, lymph node metastasis and invasion was significantly higher in the SC/ASC compared with the AC (P<0.05). It’s indicated that the clinicopathological presentations of SC/ASC might have strong invasive and metastatic potential compared to ordinary AC.

The expression of ASPH in AC and SC/ASC has not been previously reported, although their expressions have been associated with the progression and prognosis of a variety of tumors. Positive ASPH expressions are associated with TNM stages, invasion, metastasis, and poor prognosis of AC and SC/ASC. The role of ASPH expressions in gallbladder SC/ASC and AC remains to be clarified. Our study first showed that the positive expressions of ASPH were significantly higher in the cases of SC/ASC and AC than in poorly differentiation. The positive expressions of ASPH were significantly higher in the cases of poorly differentiation, large tumor size, high TNM stage, lymph node metastasis, invasion and no resection (only biopsy) of SC/ASC and AC. The univariate Kaplan-Meier analysis showed that positive ASPH expression is closely associated with a decreased overall survival in SC/ASC and AC patients. The multivariate Cox regression analysis identified that positive ASPH expression are independent factors for a poor-prognosis in SC/ASC and AC patients.

In gallbladder carcinoma, ASPH promotes the process of cell proliferation, and ASPH is mainly expressed in epithelial of gallbladder carcinoma. Apart from that, the subgroup cluster of epithelial which is the highest expression of ASPH is significant in cell cycle and adherens junction, compared with other subgroup clusters. Expression of ASPH is very low in normal adult tissues but is highly expressed in the placenta (an invasive tissue) (20). In the present, it’s found that high expression of ASPH promotes epithelial mesenchymal transition and epithelial cell migration in gallbladder cancer. Accordingly, high expression of ASPH can activate the four pathways of FN1 signaling pathway, COLLAGEN signaling pathway, VISFATIN signaling pathway and SPP1 signaling pathway, these pathways occur mainly in the epithelial, myeloid cell, myeloid cell and macrophage of gallbladder carcinoma. In previous studies, it has been demonstrated that the four pathways promotes metastasis of tumor (21–24). Its expression is “shut off” in the adult only to re-emerge during oncogenesis where it may be required for generation of malignant phenotypes (14, 25). Transcriptional regulation of ASPH is provided by tripartite signaling pathways: insulin (IN) and insulin like growth factor 1 (IGF1) and WNT/β-Catenin (26). Recent studies have demonstrated a relationship between ASPH expression and pathogenesis, progression and prognosis of some malignant lesions, the malignant lesions of overexpression of ASPH showed high malignance and poor prognosis (12–16, 27). Dong also found that ASPH is highly overexpressed in pancreatic cancer (PC) and ASPH upregulation confers a malignant phenotype characterized by enhanced cell proliferation, migration, invasion and colony formation *in vitro* as well as PC tumor growth *in vivo (* 15).

This study found that, compared to the primary lesions, liver metastases of gallbladder cancer contain a large number of immune cells. However, the liver metastases also contain a large number of exhausted CD8^+^ T cells and Treg cells, leading to T cell exhaustion in patients. Exhausted CD8^+^ cells exhibit compromised cellular activity and proliferation, elevated apoptosis rates and reduced production of effector cytokines (28). There is accumulating evidence that the removal of Treg cells is able to evoke and enhance anti-tumor immune response. Treg cells can suppress the immune response of other immune cells and are the main controller of self-tolerance. Treg cells not only control T cells through humoral and cell-cell contact mechanisms, but also B cells, NK cells, dendritic cell and macrophages (29). ASPH plays an important role in the immune abnormalities of gallbladder cancer. High expression of ASPH in gallbladder cancer promotes the migration and infiltration of immune cells, but weakens the cell recognition function of T cells and B cells, and reduces the IL1 signaling pathway, complement signaling pathway and IFN-II signaling pathway in the gallbladder cancer microenvironment. Additionally, ASPH co-expresses with PDL1 and PVR in the epithelium of gallbladder cancer, thereby achieving immune escape through common immune checkpoint pathways such as PD1 (30) and TIGIT (31). Therefore, high expression of ASPH in gallbladder cancer leads to immune therapy tolerance.

In addition to immune therapy, ASPH promotes drug metabolism through the cytochrome P450 pathway, thus enhancing drug metabolism. The metabolism regulated by cytochrome P450 isoenzymes is recognized as a significant contributor to the biotransformation of anticancer agents (32). Gemcitabine and paclitaxel are often applied to cure gallbladder cancer (33, 34). Therefore, the expression of ASPH is related to tumor resistance to chemotherapy. Tumor cells with high expression of ASPH result in a significant increase in IC50 for gemcitabine and paclitaxel. Therefore, it can be seen that ASPH can serve as a target for the treatment of gallbladder cancer. Inhibiting the expression of ASPH in gallbladder cancer can improve the treatment response in patients when used in conjunction with clinical therapy. Docking enables the identification of novel compounds of therapeutic interest, predicting ligand-target interactions at a molecular level, or delineating structure-activity relationships (SAR), without knowing *a priori* the chemical structure of other target modulators (35). Through network pharmacology and molecular docking, this study identified six traditional Chinese medicine active compounds (Sebacic acid, Suberic acid, Azelaic acid, Dimelic acid, Succinic acid, and D-Asparaginase)targeting ASPH. Future research is urgently needed to investigate these active compounds.

## 5 Conclusions

ASPH plays a role in promoting the development of gallbladder cancer and resistance to chemotherapeutic agents, rendering it a promising target for therapeutic interventions. Active therapeutic compounds targeted on ASPH can be identified among the active ingredients present in traditional Chinese medicine.

## Data Availability

The original contributions presented in the study are included in the article/**Supplementary Material**. Further inquiries can be directed to the corresponding author.

## References

[1] Jayaraman S Jarnagin WR. Management of gallbladder cancer. Gastroenterol Clinics North America. (2010) 39:331–342, x. doi: 10.1016/j.gtc.2010.02.006 20478489

[2] Jemal A Siegel R Xu J Ward E. Cancer statistics, 2010. CA: Cancer J Clin. (2010) 60:277–300. doi: 10.3322/caac.20073 20610543

[3] Reid KM Ramos-De-la-Medina A Donohue JH. Diagnosis and surgical management of gallbladder cancer: a review. J gastrointestinal Surg. (2007) 11:671–81. doi: 10.1007/s11605-006-0075-x 17468929

[4] Hawkins WG DeMatteo RP Jarnagin WR Ben-Porat L Blumgart LH Fong Y. Jaundice predicts advanced disease and early mortality in patients with gallbladder cancer. Ann Surg Oncol. (2004) 11:310–5. doi: 10.1245/ASO.2004.03.011 14993027

[5] Ootani T Shirai Y Tsukada K Muto T. Relationship between gallbladder carcinoma and the segmental type of adenomyomatosis of the gallbladder. Cancer. (1992) 69:2647–52. doi: 10.1002/1097-0142(19920601)69:11<2647::AID-CNCR2820691105>3.0.CO;2-0 1571894

[6] Park SB Kim YH Rho HL Chae GB Hong SK. Primary carcinosarcoma of the gallbladder. J Korean Surg Soc. (2012) 82:54–8. doi: 10.4174/jkss.2012.82.1.54 PMC3268145 22324048

[7] Liu DC Yang ZL. MTDH and EphA7 are markers for metastasis and poor prognosis of gallbladder adenocarcinoma. Diagn cytopathology. (2013) 41:199–205. doi: 10.1002/dc.21821 21964981

[8] Goldbrunner RH Haugland HK Klein CE Kerkau S Roosen K Tonn JC. ECM dependent and integrin mediated tumor cell migration of human glioma and melanoma cell lines under serum-free conditions. Anticancer Res. (1996) 16:3679–87. 9042241

[9] Lavaissiere L Jia S Nishiyama M de la Monte S Stern AM Wands JR. Overexpression of human aspartyl(asparaginyl)beta-hydroxylase in hepatocellular carcinoma and cholangiocarcinoma. J Clin Invest. (1996) 98:1313–23. doi: 10.1172/JCI118918 PMC507557 8823296

[10] Merzak A Koochekpour S Pilkington GJ. Adhesion of human glioma cell lines to fibronectin, laminin, vitronectin and collagen I is modulated by gangliosides in *vitro* . Cell adhesion communication. (1995) 3:27–43. doi: 10.3109/15419069509081276 7749720

[11] Tucker RP Kenzelmann D Trzebiatowska A Chiquet-Ehrismann R. Teneurins: transmembrane proteins with fundamental roles in development. Int J Biochem Cell Biol. (2007) 39:292–7. doi: 10.1016/j.biocel.2006.09.012 17095284

[12] de la Monte SM Tamaki S Cantarini MC Ince N Wiedmann M Carter JJ. Aspartyl-(asparaginyl)-beta-hydroxylase regulates hepatocellular carcinoma invasiveness. J Hepatol. (2006) 44:971–83. 10.1016/j.jhep.2006.01.038 16564107

[13] Maeda T Sepe P Lahousse S Tamaki S Enjoji M Wands JR. Antisense oligodeoxynucleotides directed against aspartyl (asparaginyl) beta-hydroxylase suppress migration of cholangiocarcinoma cells. J Hepatol. (2003) 38:615–22. doi: 10.1016/S0168-8278(03)00052-7 12713872

[14] Wang K Liu J Yan ZL Li J Shi LH Cong WM. Overexpression of aspartyl-(asparaginyl)-beta-hydroxylase in hepatocellular carcinoma is associated with worse surgical outcome. Hepatol (Baltimore Md). (2010) 52:164–73. doi: 10.1002/hep.23650 20578260

[15] Dong X Lin Q Aihara A Li Y Huang CK Chung W. Aspartate β-Hydroxylase expression promotes a Malignant pancreatic cellular phenotype. Oncotarget. (2015) 6:1231–48. doi: 10.18632/oncotarget.2840 PMC4359229 25483102

[16] Yoo HJ Yun BR Kwon JH Ahn HS Seol MA Lee MJ. Genetic and expression alterations in association with the sarcomatous change of cholangiocarcinoma cells. Exp Mol Med. (2009) 41:102–15. doi: 10.3858/emm.2009.41.2.013 PMC2679331 19287191

[17] Li L Liao J Ruland J Mak TW Cohen SN. A TSG101/MDM2 regulatory loop modulates MDM2 degradation and MDM2/p53 feedback control. Proc Natl Acad Sci United States America. (2001) 98:1619–24. doi: 10.1073/pnas.98.4.1619 PMC29306 11172000

[18] Kondo M Dono K Sakon M Shimizu J Nagano H Nakamori S. Adenosquamous carcinoma of the gallbladder. Hepato-gastroenterology. (2002) 49:1230–4. 12239911

[19] Muzio G Maggiora M Paiuzzi E Oraldi M Canuto RA. Aldehyde dehydrogenases and cell proliferation. Free Radical Biol Med. (2012) 52:735–46. doi: 10.1016/j.freeradbiomed.2011.11.033 22206977

[20] Gundogan F Elwood G Greco D Rubin LP Pinar H Carlson RI. Role of aspartyl-(asparaginyl) beta-hydroxylase in placental implantation: Relevance to early pregnancy loss. Hum Pathol. (2007) 38:50–9. doi: 10.1016/j.humpath.2006.06.005 16949909

[21] Chen C Shen Z. FN1 promotes thyroid carcinoma cell proliferation and metastasis by activating the NF-Kb pathway. Protein Pept Lett. (2023) 30:54–64. 36278453 10.2174/0929866530666221019162943

[22] Hung SY Lin CY Yu CC Chen HT Lien MY Huang YW. Visfatin promotes the metastatic potential of chondrosarcoma cells by stimulating AP-1-Dependent MMP-2 production in the MAPK pathway. Int J Mol Sci. (2021) 22(16):8642. doi: 10.3390/ijms22168642 34445345 PMC8395530

[23] Wu J Shen Y Zeng G Liang Y Liao G. SPP1(+) TAM subpopulations in tumor microenvironment promote intravasation and metastasis of head and neck squamous cell carcinoma. Cancer Gene Ther. (2024) 31:311–21. doi: 10.1038/s41417-023-00704-0 38052857

[24] Wu X Cai J Zuo Z Li J. Collagen facilitates the colorectal cancer stemness and metastasis through an integrin/PI3K/AKT/Snail signaling pathway. Biomedicine pharmacotherapy = Biomedecine pharmacotherapie. (2019) 114:108708. doi: 10.1016/j.biopha.2019.108708 30913493

[25] Ince N de la Monte SM Wands JR. Overexpression of human aspartyl (asparaginyl) beta-hydroxylase is associated with Malignant transformation. Cancer Res. (2000) 60:1261–6. 10728685

[26] Cantarini MC de la Monte SM Pang M Tong M D’Errico A Trevisani F. Aspartyl-asparagyl beta hydroxylase over-expression in human hepatoma is linked to activation of insulin-like growth factor and notch signaling mechanisms. Hepatol (Baltimore Md). (2006) 44:446–57. doi: 10.1002/hep.21272 16871543

[27] Noda T Shimoda M Ortiz V Sirica AE Wands JR. Immunization with aspartate-β-hydroxylase-loaded dendritic cells produces antitumor effects in a rat model of intrahepatic cholangiocarcinoma. Hepatol (Baltimore Md). (2012) 55:86–97. doi: 10.1002/hep.24629 PMC3242918 21898484

[28] McLane LM Abdel-Hakeem MS Wherry EJ. CD8 T cell exhaustion during chronic viral infection and cancer. Annu Rev Immunol. (2019) 37:457–95. doi: 10.1146/annurev-immunol-041015-055318 30676822

[29] Sakaguchi S Yamaguchi T Nomura T Ono M. Regulatory T cells and immune tolerance. Cell. (2008) 133:775–87. doi: 10.1016/j.cell.2008.05.009 18510923

[30] Kornepati AVR Vadlamudi RK Curiel TJ. Programmed death ligand 1 signals in cancer cells. Nat Rev Cancer. (2022) 22:174–89. doi: 10.1038/s41568-021-00431-4 PMC9989967 35031777

[31] Freed-Pastor WA Lambert LJ Ely ZA Pattada NB Bhutkar A Eng G. The CD155/TIGIT axis promotes and maintains immune evasion in neoantigen-expressing pancreatic cancer. Cancer Cell. (2021) 39:1342–60.e1314. doi: 10.1016/j.ccell.2021.07.007 34358448 PMC8511341

[32] Scripture CD Sparreboom A Figg WD. Modulation of cytochrome P450 activity: implications for cancer therapy. Lancet Oncol. (2005) 6:780–9. doi: 10.1016/S1470-2045(05)70388-0 16198984

[33] Shroff RT Javle MM Xiao L Kaseb AO Varadhachary GR Wolff RA. Gemcitabine, cisplatin, and nab-Paclitaxel for the treatment of advanced biliary tract cancers: A phase 2 clinical trial. JAMA Oncol. (2019) 5:824–30. doi: 10.1001/jamaoncol.2019.0270 PMC6567834 30998813

[34] Valle JW Furuse J Jitlal M Beare S Mizuno N Wasan H. Cisplatin and gemcitabine for advanced biliary tract cancer: a meta-analysis of two randomised trials. Ann oncology: Off J Eur Soc Med Oncol. (2014) 25:391–8. doi: 10.1093/annonc/mdt540 24351397

[35] Pinzi L Rastelli G. Molecular docking: shifting paradigms in drug discovery. Int J Mol Sci. (2019) 20(18):4331. doi: 10.3390/ijms20184331 31487867 PMC6769923

