# Supplementary figures and images for: Aspartate beta-hydroxylase is a prognostic factor in gallbladder cancer with the function of promoting tumorigenesis and chemoresistance
Source: Front Endocrinol (Lausanne). 2025 Mar 5;16:1452345. doi: 10.3389/fendo.2025.1452345 (PMC11919673; doi:10.3389/fendo.2025.1452345)

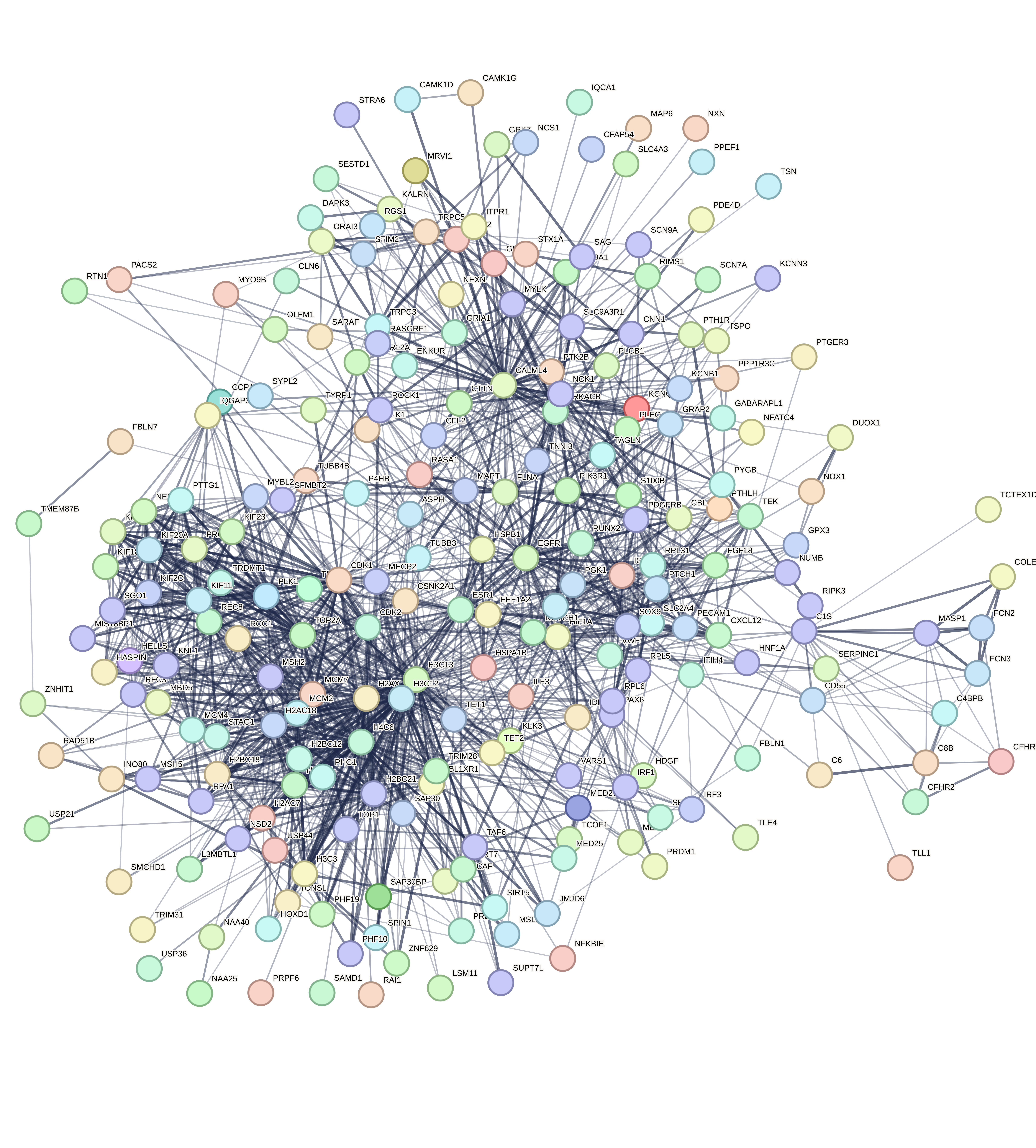

Supplement: Supplementary file 1 [file Image1.png]

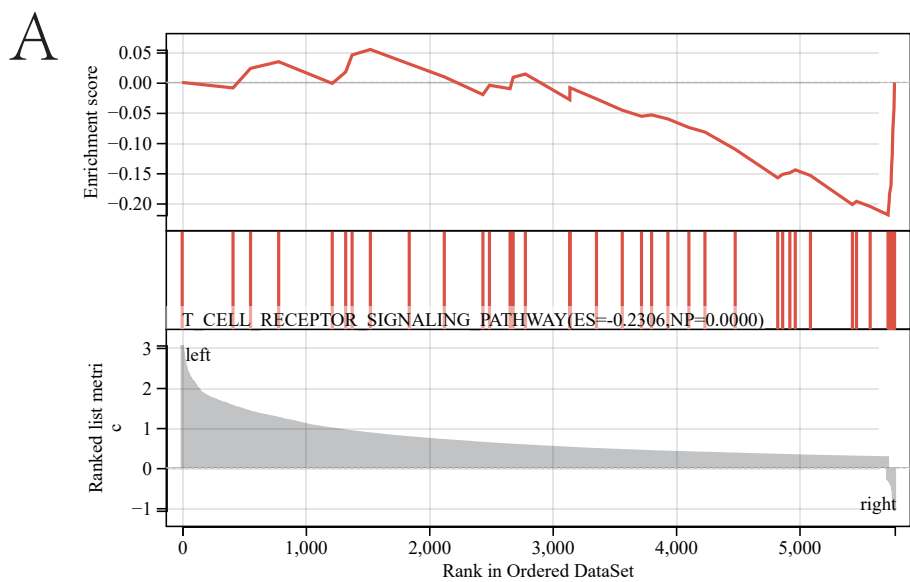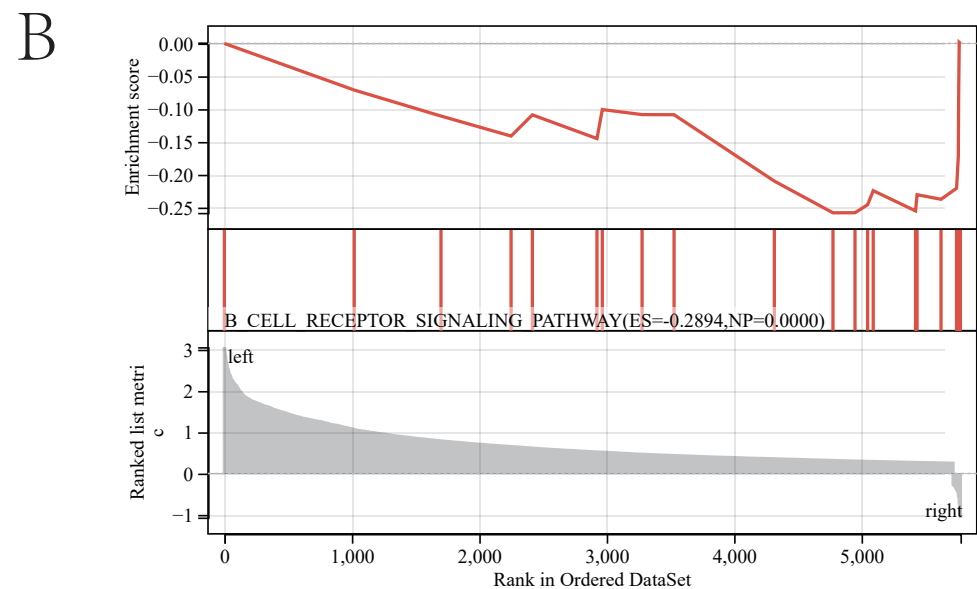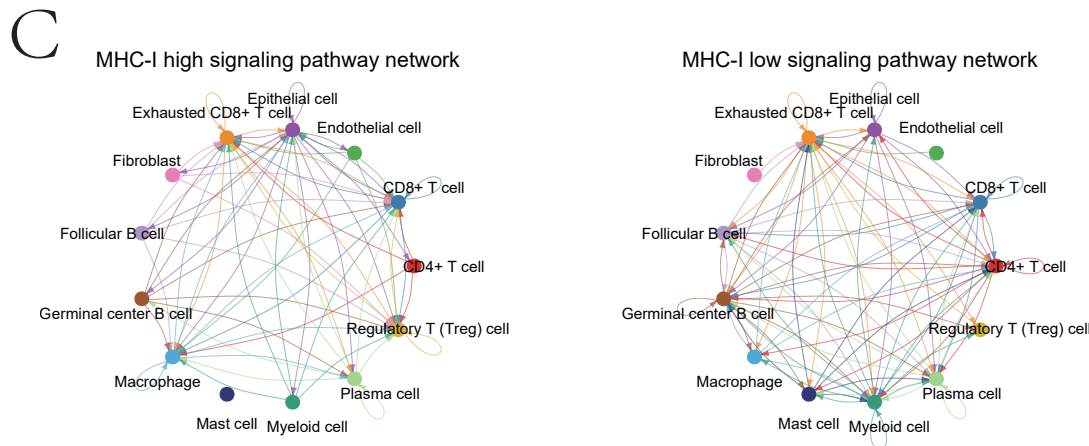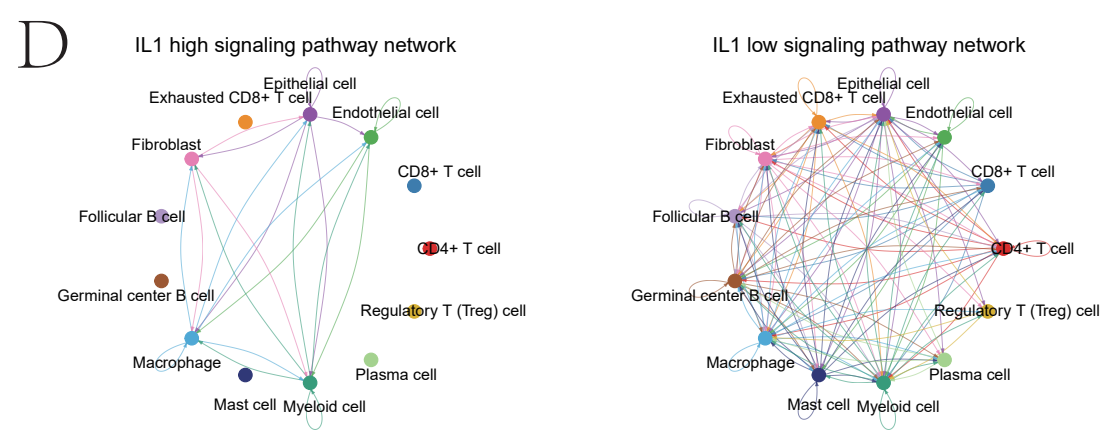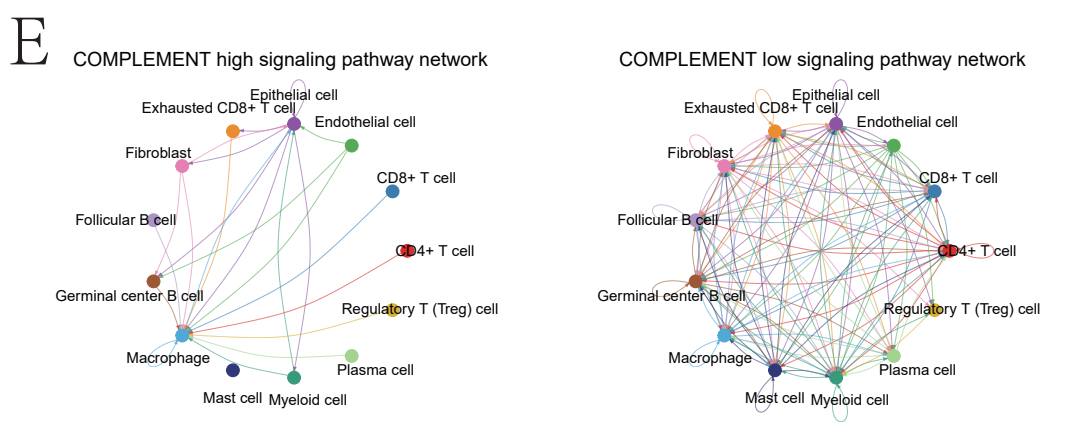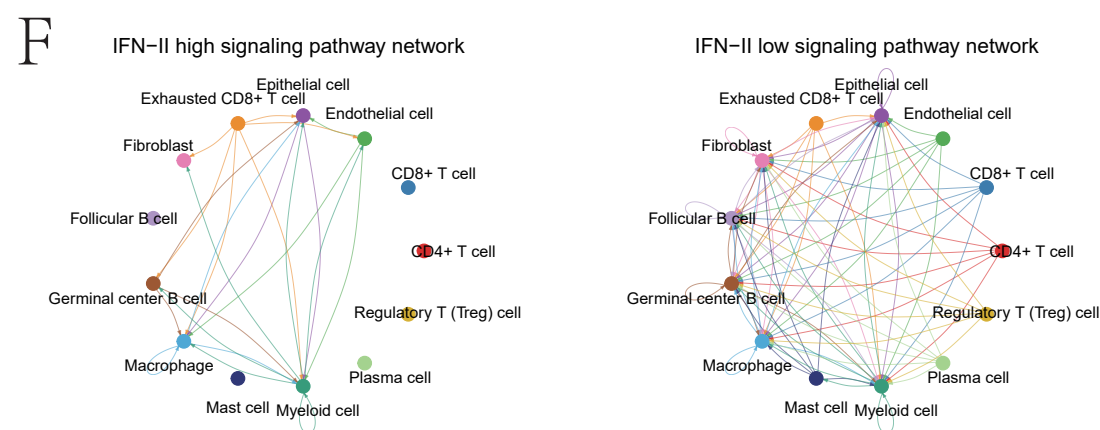

Supplement: Supplementary file 2 [file Image2.pdf]

Gallbladder cancer cell line（QBC-SD）

ASPH(84KD)


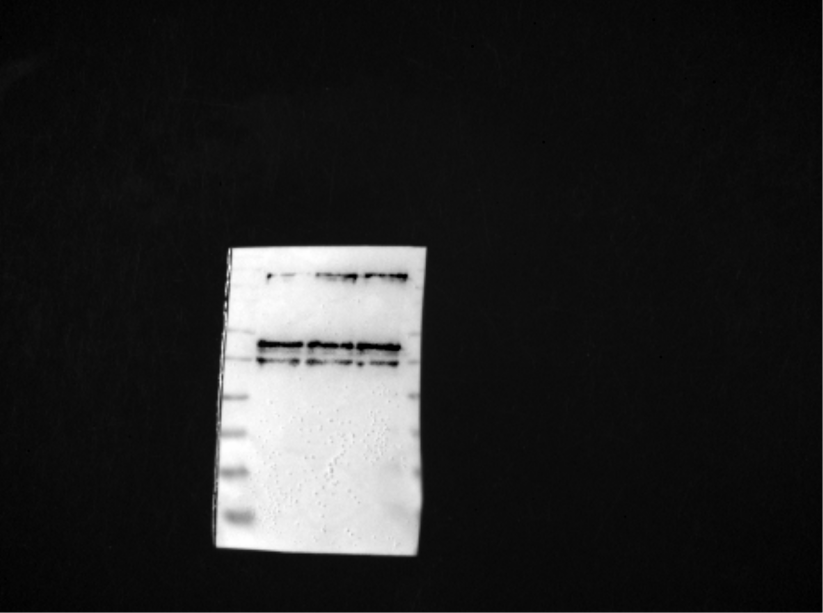


β-ACTIN(42KD)

Supplement: Supplementary file 6 [file DataSheet3.docx]
