# Supplementary material for: Aspartate beta-hydroxylase is a prognostic factor in gallbladder cancer with the function of promoting tumorigenesis and chemoresistance
Source: Front Endocrinol (Lausanne). 2025 Mar 5;16:1452345. doi: 10.3389/fendo.2025.1452345 (PMC11919673; doi:10.3389/fendo.2025.1452345)
Supplement: Supplementary file 3 [file Image3.pdf]

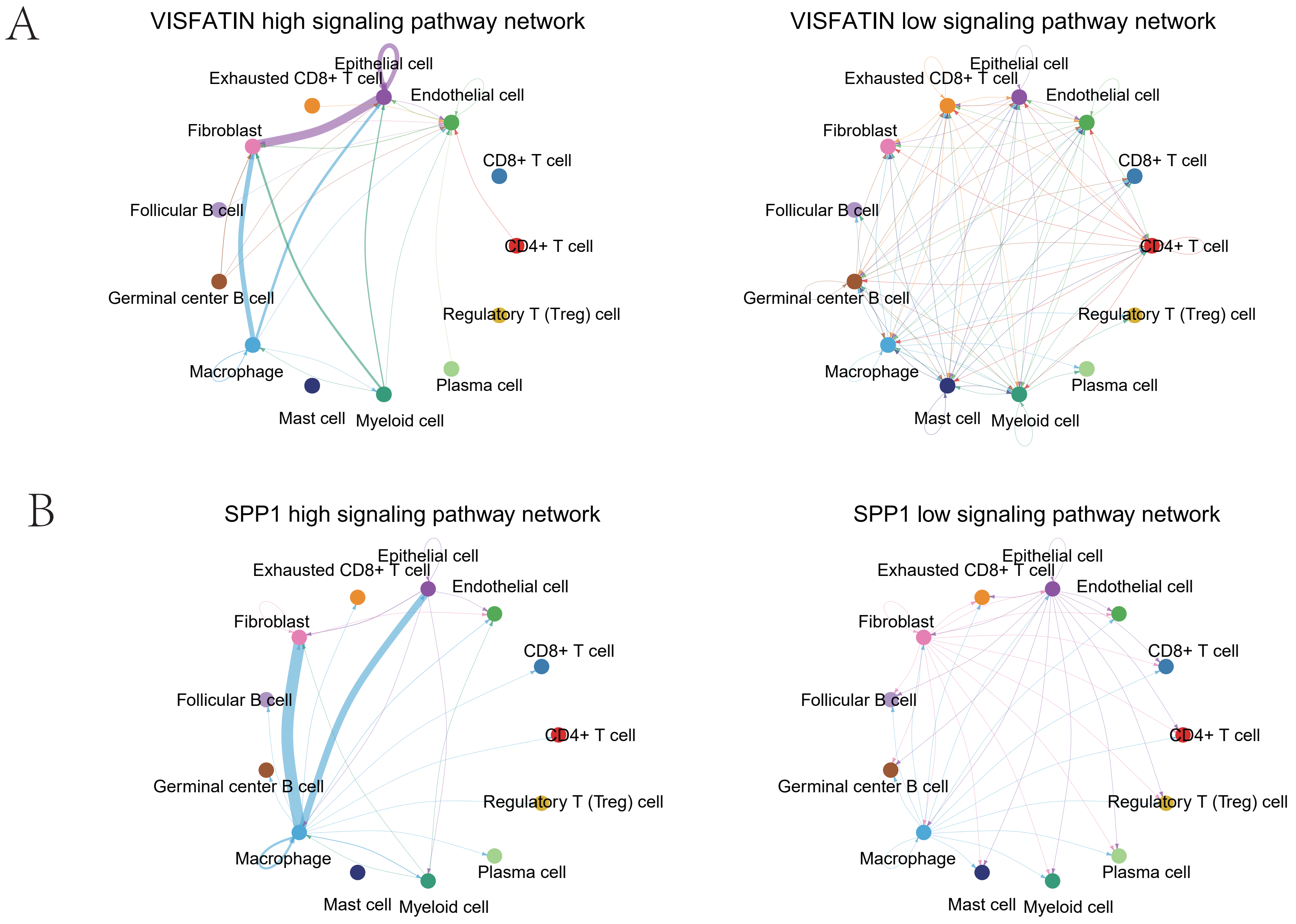
A
VISFATIN high signaling pathway network
Epithelial cell
Exhausted CD8+ T cell
Endothelial cell
Fibroblast
CD8+ T cell
Follicular B cell
CD4+ T cell
Germinal center B cell
Regulatory T (Treg) cell
Macrophage
Plasma cell
Mast cell
Myeloid cell
VISFATIN low signaling pathway network
Epithelial cell
Exhausted CD8+ T cell
Endothelial cell
Fibroblast
CD8+ T cell
Follicular B cell
CD4+ T cell
Germinal center B cell
Regulatory T (Treg) cell
Macrophage
Plasma cell
Mast cell
Myeloid cell
B
SPP1 high signaling pathway network
Epithelial cell
Exhausted CD8+ T cell
Endothelial cell
Fibroblast
CD8+ T cell
Follicular B cell
CD4+ T cell
Germinal center B cell
Regulatory T (Treg) cell
Macrophage
Plasma cell
Mast cell
Myeloid cell
SPP1 low signaling pathway network
Epithelial cell
Exhausted CD8+ T cell
Endothelial cell
Fibroblast
CD8+ T cell
Follicular B cell
CD4+ T cell
Germinal center B cell
Regulatory T (Treg) cell
Macrophage
Plasma cell
Mast cell
Myeloid cell
