# Supplementary material for: Aspartate beta-hydroxylase is a prognostic factor in gallbladder cancer with the function of promoting tumorigenesis and chemoresistance
Source: Front Endocrinol (Lausanne). 2025 Mar 5;16:1452345. doi: 10.3389/fendo.2025.1452345 (PMC11919673; doi:10.3389/fendo.2025.1452345)
Supplement: Supplementary file 7 [file Table1.doc]

**Table S1.** Relationship between ASPH expression, clinicopathological characteristics and average survival of SC/ASC and AC patients

| CPC |  | AC | | |  |  | SC/ASC | | |
| --- | --- | --- | --- | --- | --- | --- | --- | --- | --- |
|  | Case No. | ASPH Pos No. (%) | 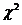 | P value |  | Case No. | ASPH Pos No. (%) | 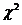 | P value |
| Differentiation |  |  |  |  |  |  |  |  |  |
| Well | 19 | 10（52.6） | 5.972 | 0.051 |  | 51 | 17（33.3） | 22.773 | 0 |
| Moderately | 33 | 19（57.6） |  |  |  | 54 | 26（48.1） |  |  |
| Poorly | 17 | 15（88.2） |  |  |  | 41 | 33（80.5） |  |  |
| Tumor size |  |  |  |  |  |  |  |  |  |
| ≤3cm | 30 | 12（40.0） | 12.978 | 0 |  | 90 | 39（43.3） | 7.151 | 0.007 |
| ＞3cm | 39 | 32（82.1） |  |  |  | 56 | 37（66.1） |  |  |
| Gallstone |  |  |  |  |  |  |  |  |  |
| No | 31 | 20(64.5) | 0.014 | 0.907 |  | 78 | 37(47.4) | 1.432 | 0.232 |
| Yes | 38 | 24(63.2) |  |  |  | 68 | 39(57.4) |  |  |
| Lymphnode metastasis |  |  |  |  |  |  |  |  |  |
| No | 27 | 10（37.0） | 13.718 | 0 |  | 80 | 35（43.8） | 4.89 | 0.035 |
| Yes | 42 | 34(81.0) |  |  |  | 66 | 41(62.1) |  |  |
| Invasion |  |  |  |  |  |  |  |  |  |
| No | 24 | 8 (33.3) | 14.753 | 0 |  | 72 | 25(34.7) | 17.099 | 0 |
| Yes | 45 | 36(80.0) |  |  |  | 74 | 51(68.9) |  |  |
| TNM stage |  |  |  |  |  |  |  |  |  |
| Ⅰ＋Ⅱ | 29 | 10﹙34.5﹚ | 18.569 | 0 |  | 77 | 29﹙37.7﹚ | 13.523 | 0 |
| Ⅲ＋Ⅳ | 40 | 34﹙85.0﹚ |  |  |  | 69 | 47﹙68.1﹚ |  |  |
| Surgery |  |  |  |  |  |  |  |  |  |
| Radical | 27 | 11（40.7） | 10.18 | 0.006 |  | 75 | 27（36.0） | 9.439 | 0.008 |
| Palliative | 28 | 22(78.6) |  |  |  | 50 | 31(62.0) |  |  |
| Biopsy | 14 | 11(78.6) |  |  |  | 21 | 18(85.7) |  |  |
