# Supplementary material for: Aspartate beta-hydroxylase is a prognostic factor in gallbladder cancer with the function of promoting tumorigenesis and chemoresistance
Source: Front Endocrinol (Lausanne). 2025 Mar 5;16:1452345. doi: 10.3389/fendo.2025.1452345 (PMC11919673; doi:10.3389/fendo.2025.1452345)
Supplement: Supplementary file 8 [file Table2.docx]

| Ingredient | Molecular Formula | Druglikeness Weight | Druglikeness Grading |
| --- | --- | --- | --- |
| Suberic Acid | C8H14O4 | 0.612 | Moderate |
| Sebacic Acid | C10H18O4 | 0.602 | Moderate |
| Succinic Acid | C4H6O4 | 0.53 | Moderate |
| Dimelic Acid | C7H12O4 | 0.608 | Moderate |
| Azelaic Acid | C9H16O4 | 0.61 | Moderate |
| D-Asparaginsaeure | C4H7NO4 | 0.367 | Weak |

Table S2. Drug-likeness of each ingredient
